# Decoding cytarabine-induced damage in the prepubertal testis: A multi-omics atlas of ecosystem and inheritance

**DOI:** 10.1016/j.isci.2026.117094

**Published:** 2026-08-05

**Authors:** Siyu Xia, Jiangpeng Wu, Jiawei Duan, Wenting Ye, Yan Sun, Xiaojian Yang, Xiao Chen, Hongru Lin, Songyu Huang, Jing Cai, Haiping Wen, Yali Song

**Affiliations:** 1 Center for Reproductive Medicine, Dongguan Key Laboratory for Innovative Research on Integrated Traditional Chinese and Western Medicine for the Diagnosis and Treatment of Infertility, Institute of Reproductive Genetics, Dongguan Maternal and Child Health Care Hospital, Dongguan, Guangdong Province 523000, China

**Keywords:** Cytarabine (Ara-C), Testicular Toxicity, Fertility Preservation, Multi-Omics, Spermatogonial Stem Cells

## Abstract

Pediatric leukemia therapies can compromise future fertility, particularly in prepubertal boys who cannot bank sperm. We used dose-response histology, endocrine and sperm analyses, bulk and single-cell RNA-seq, and metabolomics to map cytarabine (Ara-C) injury in prepubertal mouse testes. Ara-C caused seminiferous-tubule disruption, hormone suppression, sperm loss, spermatogonial depletion linked to ferroptosis and replication arrest, stress remodeling of Sertoli and Leydig cells, and altered intercellular signaling centered on Sertoli hubs. Unexposed F1 offspring retained inflammatory, nucleotide, and energy-metabolism changes, suggesting intergenerational imprinting after paternal exposure. These findings define cell-type-specific vulnerabilities in the developing testis and nominate redox balance, ferroptosis, Sertoli stress pathways, Leydig inflammatory signaling, and niche communication as targets for fertility-preserving strategies during pediatric chemotherapy.

## Highlights

• Ara-C disrupts prepubertal testis architecture, hormones, and sperm output
• Single-cell RNA-seq maps germ and somatic vulnerabilities after Ara-C
• Ferroptosis and replication arrest deplete spermatogonia
• F1 testes retain inflammatory and metabolic signatures after paternal exposure

## Graphical abstract

**Figure:**
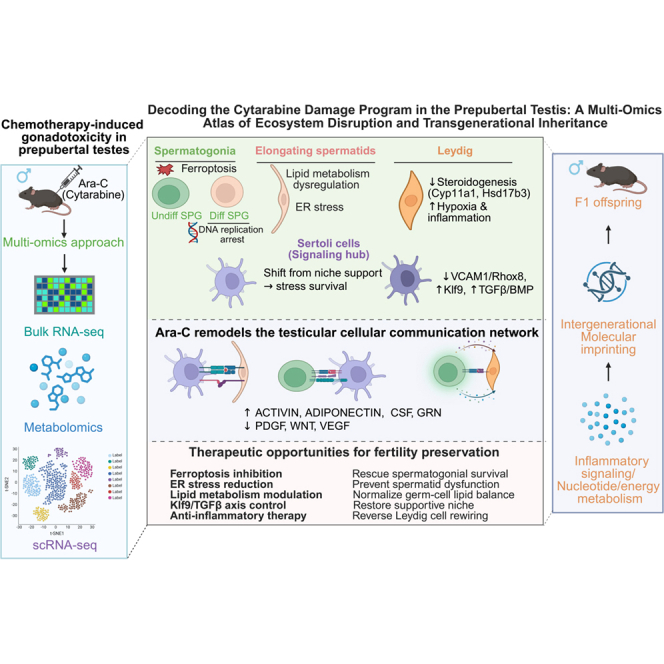

## Teaser

Biological sciences; Omics

## Introduction

In recent decades, treatment outcomes for pediatric and adolescent cancer patients have improved substantially, with the overall five-year survival rate rising from approximately 58% in the late 1970s to around 80% today.^1^^,^^2^ This remarkable achievement is closely linked to, and inseparable from, the continuous optimization of chemotherapy regimens, together with advances in diagnostic techniques, supportive care, and multidisciplinary treatment strategies,^3^^,^^4^ such as risk-adapted protocols, surgery combined with radiotherapy when indicated, hematopoietic stem cell transplantation, and integrated supportive care delivered by multidisciplinary teams.^5^ However, the growing population of long-term survivors has brought new challenges, among which fertility preservation has emerged as a key concern.^6^ Chemotherapeutic agents, while effectively targeting malignant cells, also exert non-specific cytotoxicity on healthy tissues.^7^ Gonadotoxicity represents one of the most frequent and persistent long-term adverse effects,^8^ and is particularly critical in prepubertal boys who have not yet initiated spermatogenesis and therefore cannot cryopreserve sperm as adult patients can.^9^^,^^10^ In addition, prepubertal testicular tissue contains a high proportion of proliferating and differentiating germ cells, rendering it especially vulnerable to chemotherapy-induced damage.^11^^,^^12^ Taken together, these factors underscore the challenges of fertility preservation in pediatric patients, and even in pubertal boys, sperm cryopreservation may not always be feasible due to collection failure, making testicular tissue cryopreservation a potential alternative strategy.^13^

Basic research has offered promising strategies for fertility restoration following gonadotoxic treatments. In animal models, transplantation of spermatogonial stem cells (SSCs) or immature testicular tissue has successfully generated functional gametes and produced healthy offspring.^10^ Furthermore, *in vitro* spermatogenesis has advanced to early stages in both mouse and human testicular tissue, although completion of the full process in humans has yet to be achieved.^14^^,^^15^ A recent study reported that three-dimensional culture of testicular biopsy tissue from chemotherapy-treated prepubertal boys induced SSC differentiation into sperm-like cells. While this finding represents a significant step toward clinical translation, success was achieved in only one of six samples, and the functional competence of these sperm-like cells remains to be validated.^16^ Beyond low efficiency, *in vitro* culture of human testicular tissue generally yields disappointing success rates.^17^ For patients with non-solid tumors such as leukemia, malignant cell infiltration of the testes further increases the risk of disease recurrence upon autologous transplantation, a concern substantiated by xenotransplantation studies demonstrating leukemic cell contamination in testicular tissue.^18^

The mammalian testis is a highly specialized organ responsible for spermatogenesis and steroidogenesis, processes that are indispensable for male fertility and the maintenance of systemic endocrine and metabolic homeostasis.^15^ Spermatogenesis is tightly orchestrated, encompassing SSC self-renewal and differentiation, meiotic division, and spermiogenesis. Each stage depends on a finely tuned somatic cell niche, particularly Sertoli cells, Leydig cells and peritubular myoid cells, which provide essential structural and biochemical support.^19^ This complex cellular network supporting spermatogenesis is highly susceptible to environmental insults, including chemotherapeutic agents.^20^ Cryopreservation of immature testicular tissue prior to treatment has been proposed as an experimental strategy aimed at restoring fertility in the future via transplantation or *in vitro* culture.^21^ The success of this approach fundamentally depends on the preserved tissue containing sufficient numbers of morphologically and functionally intact SSCs.^11^ An alternative strategy involves administering protective interventions before or during chemotherapy to prevent gonadal damage at its source.^22^ Developing agents that selectively protect the prepubertal testis without compromising anticancer efficacy offers a promising direction, yet requires a thorough understanding of drug-specific injury mechanisms.

Cytarabine (Ara-C), a cornerstone agent in leukemia chemotherapy widely used in pediatric patients including prepubertal boys,^23^ is a nucleoside analog that interferes with DNA synthesis in rapidly proliferating cells and induces gonadotoxicity as one of its most frequent long-term complications.^24^ This poses a particular clinical challenge for prepubertal patients who lack the option of sperm cryopreservation due to absent spermatogenesis.^25^ Although clinical phenotypes such as oligozoospermia/azoospermia and bulk omics studies have indicated testicular damage,^26^^,^^27^ the precise cellular mechanisms underlying Ara-C-induced testicular toxicity remain largely undefined, especially in the developing prepubertal testis.^12^ Most existing studies are limited to histological alterations or bulk transcriptomic analysis without resolving cell-type-specific responses.^19^ Elucidating the mechanisms at single-cell resolution underlying prepubertal testicular injury induced by Ara-C is imperative for optimizing fertility preservation strategies and developing effective protective interventions.

Here we combine bulk and single-cell RNA-seq with metabolomics to chart Ara-C induced remodeling of the prepubertal mouse testis. We show dose dependent architectural and endocrine injury with sperm loss; spermatogonial depletion via ferroptosis and selective arrest of DNA replication programs; a Sertoli cell switch from niche support to KLF9 linked stress survival with TGFβ/bone morphogenetic protein (BMP) activation; Leydig cell downshifting of steroidogenesis alongside hypoxia and inflammation associated signaling; and a hyperconnected niche centered on Sertoli hubs. Unexposed F1 offspring retain discrete transcriptomic and metabolic alterations enriched for inflammatory and nucleotide and energy pathways, indicating intergenerational imprinting. This framework is designed to identify vulnerable lineages and actionable pathways that can inform fertility-preserving interventions without compromising antileukemic efficacy. Accordingly, this atlas nominates tractable targets including ferroptosis, endoplasmic reticulum (ER) stress and lipid handling pathways, the KLF9 and TGFβ axis in Sertoli cells, and inflammatory rewiring in Leydig cells for fertility preservation in pediatric oncology.

## Results

### Dose dependent testicular toxicity and persistent molecular reprogramming following prepubertal Ara-C exposure

**Figure 1:**
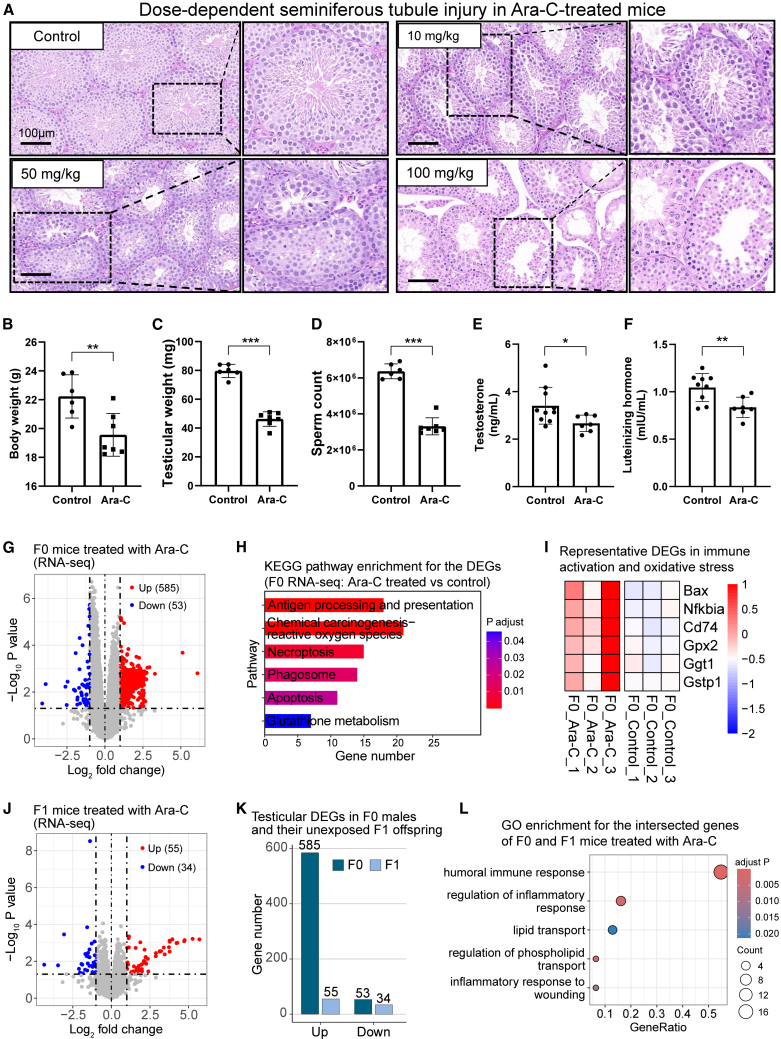
Dose-dependent testicular toxicity and transcriptomic reprogramming following prepubertal Ara-C exposure (A) Representative H&E staining of testicular sections from 2-week-old male mice treated with Ara-C (0, 10, 50, or 100 mg/kg b.w.) for four weeks, showing progressive seminiferous tubule injury in a dose-dependent manner. Shown are representative images from each group (*n* = 3 biologically independent animals). Scale bars, 100 μm. (B)–(F) Morphometric and hormonal alterations in the 100 mg/kg Ara-C group: (B) body weight, (C) bilateral testis weight, (D) total sperm count (*n* = 6 for control, *n* = 7 for Ara-C), and serum levels of (E) testosterone (*n* = 10 for control, *n* = 7 for Ara-C) and (F) luteinizing hormone (LH) (*n* = 9 for control, *n* = 7 for Ara-C) were all significantly reduced compared with controls, indicating endocrine disruption and spermatogenic failure. Data are presented as mean ± SEM. ∗*p* < 0.05; ∗∗*p* < 0.01; ∗∗∗*p* < 0.001; ∗∗∗∗*p* < 0.0001 vs. control group (Student’s *t* test). (G) Volcano plot of differentially expressed genes (DEGs) in testes from F0 mice treated with 100 mg/kg Ara-C (*n* = 3 biologically independent samples per group). Red and blue dots represent 585 upregulated and 53 downregulated genes, respectively. (H) Bar plot of KEGG pathway enrichment analysis for the DEGs. The bar length represents the number of genes, and the color intensity (blue to red) indicates the level of statistical significance (-log_10_ (adjusted *p* value)). (I) Heatmap of the expression of representative DEGs (*Cd74*, *Nfkbia* and *Gpx2*) involved in immune activation and oxidative stress. The color scale (blue to red) indicates the relative gene expression level. (J)–(K) Analysis of DEGs in the F1 generation. (J) Volcano plot and (K) bar chart showing 89 DEGs (55 upregulated in red, 34 downregulated in blue) identified in testes of F1 offspring (*n* = 3 per group), which were not directly exposed to Ara-C, derived from F0 males that retained partial fertility following Ara-C exposure. (L) Dot plot of GO pathway enrichment for DEGs shared between F0 and F1 generations, highlighting persistent activation of inflammatory and lipid metabolism pathways. The dot color represents the adjusted *p* value, and the dot size represents the number of enriched genes. Asterisks denote statistical significance: ∗*p* < 0.05; ∗∗*p* < 0.01; ∗∗∗*p* < 0.001; ∗∗∗∗*p* < 0.0001.

To assess the prepubertal testicular toxicity of cytarabine (Ara-C), two-week-old male mice received daily intraperitoneal injections of Ara-C (0, 10, 50, or 100 mg/kg body weight in phosphate-buffered saline) for four weeks. Histological analysis demonstrated dose-dependent seminiferous tubule injury (Figure 1A). Control tubules displayed intact architecture with normal germ cell stratification and interstitial structure. The 10 mg/kg group showed mild changes, including slightly reduced tubule diameter and sporadic nuclear pyknosis observed in scattered germ cells. At 50 mg/kg, the injury intensified, featuring epithelial vacuolization, disorganized germ cells, and interstitial edema. The highest dose (100 mg/kg) induced severe damage, characterized by extensive germ cell loss, basement membrane rupture, and marked edema. Collectively, these findings reveal a progressive, dose-related pattern of germ cell depletion and structural disruption.

Consistent with the histopathological deterioration, the high-dose (100 mg/kg) group exhibited significant reductions in body weight (Figure 1B), bilateral testis weight (Figure 1C), and total sperm count (Figure 1D). Serum testosterone and luteinizing hormone levels were also markedly suppressed (Figures 1E and 1F), indicating that Ara-C not only causes structural damage to spermatogenesis but also disrupts the endocrine milieu necessary for testicular maturation.

Morphological examination of the epididymis revealed corresponding changes (Figures S1A and S1B). Control epididymal tubules displayed an intact structure with well-organized columnar epithelial cells and compact, continuous walls. The lumens were dilated and densely packed with mature spermatozoa. In contrast, the Ara-C-treated group exhibited mild exfoliation and degeneration of the epididymal epithelium, accompanied by a marked reduction in sperm density and the occasional presence of cellular debris within the lumens. H&E staining confirmed a significant decrease in sperm count in the Ara-C group.

To investigate the underlying molecular mechanisms, we performed bulk RNA sequencing (RNA-seq) on testicular tissues from founder generation (F0) mice treated with 100 mg/kg Ara-C and their untreated F1 offspring. Principal component analysis (PCA) revealed distinct gene expression profiles between control and treated groups in both the F0 and F1 generations (Figure S1C). Transcriptomic analysis identified 618 differentially expressed genes (DEGs) in the F0 generation, comprising 585 upregulated and 33 downregulated genes (Figure 1G). Kyoto Encyclopedia of Genes and Genomes (KEGG) pathway enrichment analysis highlighted the significant involvement of these DEGs in key biological processes, including antigen processing and presentation, chemical carcinogenesis-reactive oxygen species, apoptosis, and glutathione metabolism (Figure 1H). This expression profile reflects pronounced immune/inflammatory activation, oxidative stress, and cell death within the testicular microenvironment. Notably, immune/inflammatory mediators such as *Cd74* and *Nfkbia*, along with the oxidative stress-related gene *Gpx2*, were significantly upregulated (Figure 1I), providing molecular support for the observed germ cell injury.

Even in the absence of direct Ara-C exposure, testes from the F1 offspring exhibited 89 DEGs (55 upregulated and 34 downregulated) compared to controls (Figures 1J and 1K). These genes were primarily enriched in signaling pathways related to inflammatory response and immune processes (Figure S1D). GO pathway enrichment analysis of the DEGs shared between F0 and F1 generations highlighted pathways involved in inflammatory response and lipid transport/metabolism (Figure 1L). Several genes, including *Apoa1*, *Ccl5*, *Lgals2*, *Reg3b*, and *Reg3g*, showed consistent dysregulation across both generations. Importantly, immune-related genes such as *Apoa1* and *Ccl5* were persistently altered in the F1 generation (Figure S1E). The downregulated genes (Figure S1F), such as *Gm24182*, *Gm25721*, *Gm45090*, and *Rps13ps1*, were predominantly pseudogenes or encoded ribosomal proteins. This decreased expression suggests impaired ribosomal function, which could impact protein synthesis and spermatogenesis.

### Single-cell transcriptomic profiling identifies cell type-specific compositional shifts and distinct transcriptional response patterns under Ara-C exposure

**Figure 2:**
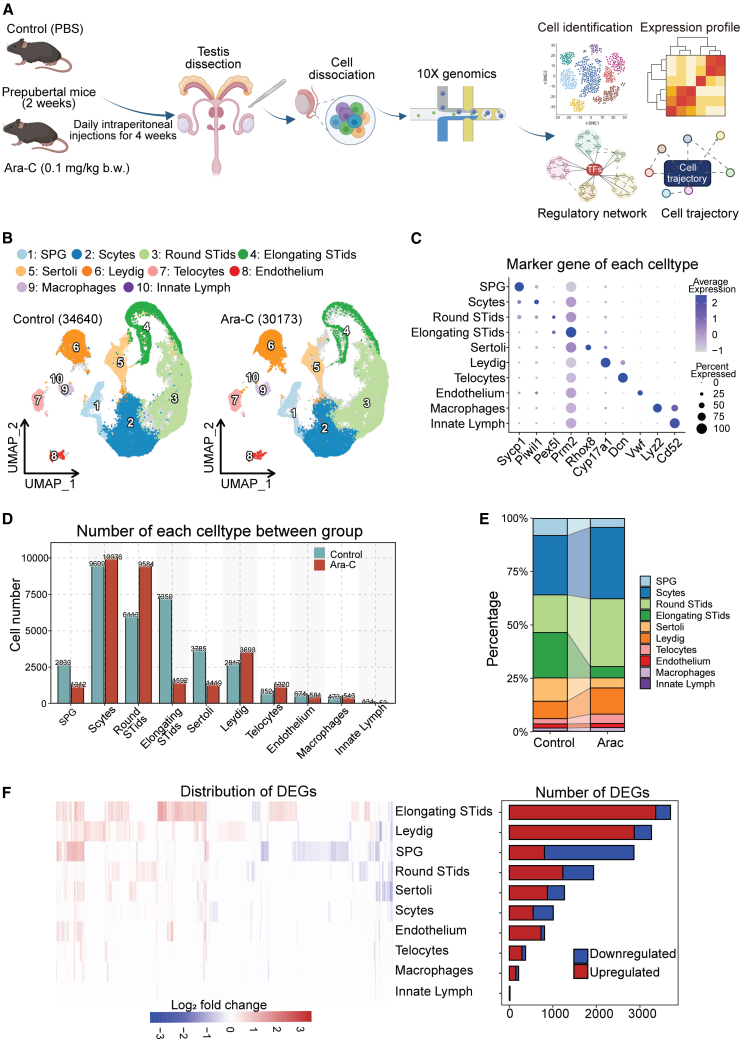
Single-cell transcriptomic profiling reveals cell type-specific compositional shifts and transcriptional response patterns following prepubertal Ara-C exposure (A) Experimental workflow of single-cell RNA-seq analysis of testes from control and Ara-C-treated (100 mg/kg b.w.) mice (samples pooled from *n* = 3 mice per group). (B) UMAP visualization of 60,619 single-cell transcriptomes (control = 34,360; Ara-C = 26,259) identifying nine major testicular cell types: spermatogonia (SPG), meiotic spermatocytes (Scytes), round spermatids (round STids), elongating spermatids (elongating STids), Sertoli cells, Leydig cells, innate lymphocytes, telocytes, and macrophages. (C) Dot plot validating cell-type annotation using canonical marker genes. Dot size represents the percentage of cells with non-zero expression for each gene prior to scaling, while color intensity indicates the scaled average expression (*Z* score) across cell types. (D) and (E) Bar plots illustrating cell type-specific compositional changes under Ara-C exposure. Bar height represents the absolute cell number (D) or the relative proportion (E) of each cell population within the sample. SPG and Sertoli cells markedly declined, whereas Scytes and round STids increased, indicating developmental arrest at mid- and late-spermatogenic stages. (F) Analysis of cell type-specific transcriptional responses. Left: Heatmap depicting the number of differentially expressed genes (DEGs, red for upregulated, blue for downregulated, Wilcoxon signed-rank test, adjusted *p* < 0.05, |Log_2_FC| > 0.25) across testicular cell types. Right: Bar plot showing the total number of DEGs identified in each cell type.

To pinpoint the specific cellular targets and mechanisms underlying the Ara-C-induced testicular toxicity observed in our bulk transcriptomic data, we performed single-cell RNA sequencing (scRNA-seq) on testicular tissues from control and Ara-C-treated (100 mg/kg) mice (Figure 2A). Before quality control, a total of 66,730 cells were profiled. After quality control (Figures S2A–S2D), we obtained 60,619 high-quality single-cell transcriptomes (control: 34,360; Ara-C: 26,259). Unsupervised clustering based on uniform manifold approximation and projection (UMAP) visualization resolved nine distinct cell types: spermatogonia (SPG), meiotic spermatocytes (Scytes), round spermatids (round STids), elongating spermatids (elongating STids), Sertoli cells, Leydig cells, innate lymphocytes, telocytes, and macrophages (Figure 2B). Cell-type annotation was validated by canonical marker gene expression patterns^28^^,^^29^^,^^30^; both the mean expression levels and the proportion of expressing cells were highly specific and consistent across clusters (Figures 2C and S2E), ensuring robust classification and enabling downstream pathway-level interpretation.

Compositional analysis of the 60,619 identified cells used for statistical analysis uncovered that Ara-C exposure led to a profound reorganization of the testicular cellular repertoire (Figures 2D, 2E, and S2F). SPG displayed concurrent declines in both relative proportion and absolute cell number, indicating depletion of the germline progenitor pool. In contrast, Scytes and round STids both increased in relative and absolute abundance, suggesting developmental arrest and cellular accumulation at these mid- and late-spermatogenesis stages. The decline in both the proportion and absolute number of elongating STids corresponded to a failure to generate mature spermatozoa. Sertoli cells also decreased in both metrics, pointing to microenvironmental support impairment. Furthermore, innate lymphocytes were reduced, implying altered testicular immune composition (Table S1).

Differential gene expression profiling across cell types exposed further layers of heterogeneity in the Ara-C response (Table S2). SPG exhibited the largest fold-change magnitude for downregulated genes (many with log_2_ fold change >2), and had few upregulated genes, confirming global transcriptional suppression in this lineage. Conversely, elongating STids showed strong upregulation with a comparable fold-change magnitude, reflecting an opposite transcriptional direction. Leydig cells, while displaying a substantial number of DEGs, also exhibited primarily upregulation but with less extreme fold changes (Figures 2F and S2G). Quantitative DEG comparisons revealed that elongating STids, Sertoli cells, and Leydig cells harbored the highest counts of upregulated genes (>2000 DEGs in elongating STids), whereas SPG was dominated by downregulated genes (Table S3).

### Ara-C induces cell type-specific transcriptional and metabolic reprogramming in germ cell subpopulations

**Figure 3:**
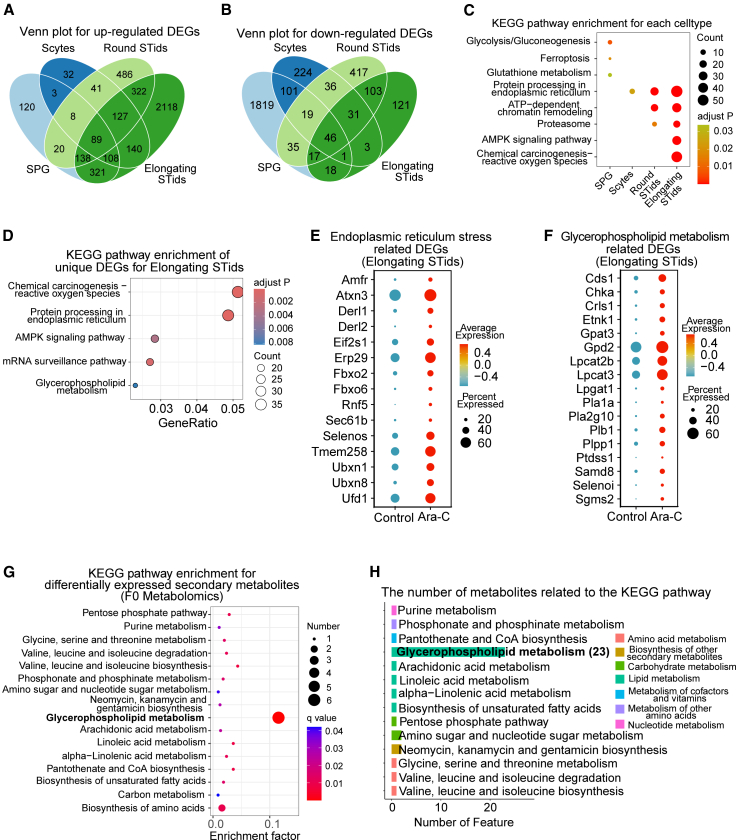
Ara-C induces cell type-specific transcriptional and metabolic reprogramming in germ cell subpopulations (A) and (B) Venn diagrams illustrating the limited overlap of differentially expressed genes (DEGs) among germ cell subtypes under Ara-C exposure. Only a small subset of DEGs (89 upregulated and 46 downregulated) was shared across all subtypes, while the majority were cell-type specific. SPG displayed predominant transcriptional suppression (1,819 downregulated and 120 upregulated genes), whereas elongating STids exhibited the opposite trend (2,118 upregulated and 121 downregulated genes). (C) and (D) Dot plots showing KEGG pathway enrichment of DEGs in SPG and elongating STids. SPG DEGs were enriched in ferroptosis, glycolysis, and glutathione metabolism pathways, suggesting redox imbalance and lipid peroxidation-associated cell death. In contrast, elongating STids were enriched for endoplasmic reticulum (ER) stress, oxidative stress, AMPK signaling, mRNA surveillance, and triglyceride metabolism pathways. Dot color intensity represents the adjusted *p* value, and dot size represents the number of enriched DEGs. (E) and (F) Heatmaps showing expression patterns of representative genes involved in ER stress and triglyceride metabolism, indicating broad activation of these pathways in elongating STids following Ara-C exposure. (G) and (H) Dot plot showing metabolic pathway enrichment of Ara-C-altered metabolites. Dot size represents the number of metabolites, and dot color intensity indicates the adjusted *p* value.

Differential gene expression analysis across germ cell subpopulations revealed that Ara-C induced both shared and highly cell type-specific transcriptional responses (Figures 3A and 3B). Only a small subset of DEGs was common to all subtypes (89 upregulated and 46 downregulated), whereas the majority were unique to specific populations. Spermatogonia displayed the most extensive transcriptional suppression, with 1,819 uniquely downregulated genes and 120 uniquely upregulated genes, while elongating spermatids (elongating STids) exhibited the opposite pattern—2,118 uniquely upregulated and 121 uniquely downregulated genes. Round spermatids (round STids) and meiotic spermatocytes (Scytes) showed smaller numbers of unique DEGs. This asymmetry is consistent with compositional analyses showing that SPG and elongating STids underwent the largest proportional changes under Ara-C exposure, indicating their high susceptibility to transcriptional and functional disruption.

Pathway enrichment analysis underscored divergent biological responses of SPG and elongating STids (Figures 3C and 3D). In SPG, DEGs were predominantly enriched in ferroptosis, glycolysis, and glutathione metabolism pathways, implicating redox imbalance and lipid peroxidation-associated cell death in progenitor depletion. Conversely, enrichment in elongating STids highlighted processes involved in ER stress, oxidative stress, AMP-activated protein kinase (AMPK) signaling, and triglyceride metabolism, together with mRNA surveillance (Tables S4 and S5). Consistent with recent findings that Ara-C primarily disrupts DNA double-strand break repair,^23^ gene set scores analysis revealed inhibition of this process in both SPG and elongating STids, with a polarized distribution of repair-related gene expression (Figures S3A and S3B). The widespread activation of ER stress and triglyceride metabolism-related genes (Figures 3E and 3F) support a model in which Ara-C impairs lipid homeostasis and protein quality control during sperm maturation.

Given the enrichment of transcriptional programs responsive to Ara-C in metabolic and redox pathways, we investigated whether these changes were accompanied by transgenerational metabolic remodeling. Metabolomic profiling of F0 and F1 generations (Figure S3C) confirmed robust metabolic changes induced by Ara-C in both, clearly separating the treatment groups from controls. However, the scope of remodeling differed markedly between generations. The F0 generation exhibited broad shifts across lipid, amino acid, organic acid, and nucleotide classes. In contrast, F1 alterations were narrower, primarily concentrated in nucleotides and energy-related compounds. Pathway analysis (Figure S3D) reinforced this dichotomy: F0 responses involved glycerophospholipid, purine, branched-chain amino acid, arachidonic acid, and amino sugar metabolism, indicating multi-pathway reprogramming. The F1 generation, however, showed enrichment largely in purine metabolism and oxidative phosphorylation, suggesting a focused response on energy and genetic information processing. Consistently, heatmap analyses showed widespread disruptions in membrane lipids, amino acids/dipeptides, and nucleotides in F0 testes, while F1 changes were mainly confined to nucleotide derivatives (Figures S3E–S3G). Further pathway analysis of altered metabolites (Figures 3G and 3H) supported the transcriptomic data, linking changes induced by Ara-C to triglyceride metabolism. This signature aligned with the upregulated lipid metabolic pathways in elongating STids, revealing a coordinated transcriptional-metabolic reprogramming after Ara-C exposure.

### Ara-C differentially impairs undifferentiated and differentiated spermatogonia

**Figure 4:**
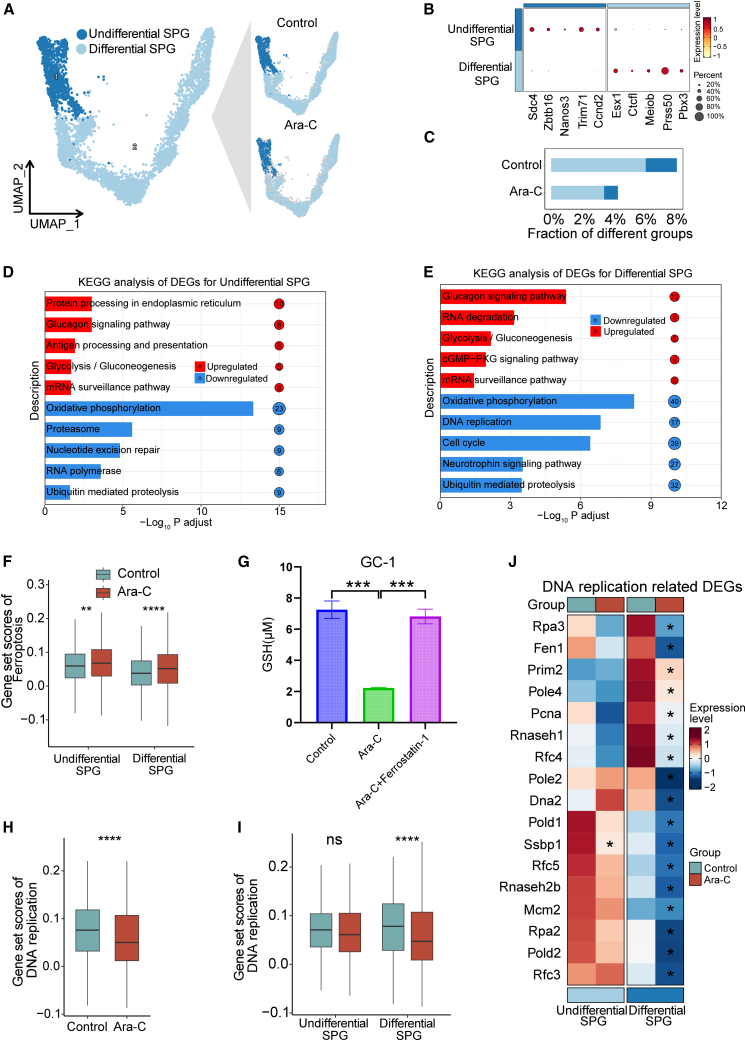
Ara-C differentially impairs undifferentiated and differentiated spermatogonia (A) UMAP plot showing reclustering of the spermatogonial (SPG) compartment from control and Ara-C-treated (100 mg/kg b.w.) testes, resolving two transcriptionally distinct states. (B) Feature plots showing marker gene expression distinguishing undifferentiated SPG (*Zbtb16*, *Sdc4*) and differentiated SPG (*Esx1*, *Ctcfl*). (C) Bar plot quantifying cell abundance, showing significant reductions in both SPG clusters, with undifferentiated SPG displaying greater depletion, indicating preferential sensitivity of early germline progenitors to Ara-C. (D) and (E) Bar plots showing KEGG pathway enrichment of differentially expressed genes (DEGs) in undifferentiated (D) and differentiated (E) SPG. Both clusters exhibited activation of glycolysis and glutathione metabolism but repression of oxidative phosphorylation, suggesting mitochondrial dysfunction and redox imbalance. (F) Gene set score analysis for ferroptosis across the SPG compartment revealed globally elevated activity after Ara-C exposure. Statistical analysis by two-sided Wilcoxon rank-sum test: ∗*p* < 0.05; ∗∗*p* < 0.01; ∗∗∗*p* < 0.001; ∗∗∗∗*p* < 0.0001 vs. control. (G) Bar plot showing that glutathione (GSH) levels were significantly suppressed upon treatment with Ara-C, validating ferroptosis activation in SPG, whereas Ferrostatin-1 (Fer-1), a potent inhibitor of ferroptosis, partially reversed these changes. Data are presented as mean ± SEM (*n* = 6 independent replicates). Statistical significance determined by Student’s *t* test: ∗*p* < 0.05; ∗∗*p* < 0.01; ∗∗∗*p* < 0.001; ∗∗∗∗*p* < 0.0001 vs. control. (H) and (I) Gene set score analysis revealed suppression of DNA replication programs in total SPG, which was not significant in undifferentiated SPG but was markedly suppressed in differentiated SPG. Statistical analysis by two-sided Wilcoxon rank-sum test: ∗*p* < 0.05; ∗∗*p* < 0.01; ∗∗∗*p* < 0.001; ∗∗∗∗*p* < 0.0001 vs. control. (J) Heatmap showing downregulation of DNA replication-related genes (*PCNA, Rnaseh1, Fen1, Ssbp1*) predominantly in differentiated SPG, indicating impaired proliferative capacity. Statistical analysis by two-sided Wilcoxon rank-sum test: ∗*p* < 0.05; ∗∗*p* < 0.01; ∗∗∗*p* < 0.001; ∗∗∗∗*p* < 0.0001 vs. control.

Single-cell transcriptomic analysis revealed a pronounced depletion of SPG following Ara-C exposure. To define underlying heterogeneity, we re-clustered the SPG compartment (Figure 4A) and resolved two distinct transcriptional states: an undifferentiated SPG characterized by *Zbtb16* and *Sdc4*, and a differentiated SPG defined by *Esx1* and *Ctcfl* (Figure 4B). Quantification of cell abundance showed a significant decrease in both clusters, with undifferentiated SPG exhibiting more severe loss (Figures 4C; Table S6), suggesting that Ara-C preferentially affects early germline progenitors. Differential expression analysis identified 302 upregulated and 294 downregulated genes shared between the two SPG subtypes, while most DEGs were unique, reflecting heterogeneous transcriptional responses (Figures S4A and S4B).

KEGG pathway enrichment analysis revealed activation of glycolysis and glutathione metabolism and repression of oxidative phosphorylation in both SPG clusters (Tables S7 and S8), indicating metabolic reprogramming accompanied by mitochondrial dysfunction and redox imbalance (Figures 4D and 4E). Such changes often predispose cells to oxidative stress-related death mechanisms, and gene set enrichment analysis (GSEA) of bulk RNA-seq data revealed significant activation of one such pathway, ferroptosis, in Ara-C-treated testes (Figure S4C), consistent with iron-dependent lipid peroxidation-driven cell death. Mapping ferroptosis pathway activity across SPG confirmed a global activation pattern (Figures S4D and S4E). Gene set scores analysis further demonstrated ferroptosis activation in both undifferentiated and differentiated SPG (Figure 4F). To establish a causal link between ferroptosis and Ara-C-induced spermatogonial loss, GC-1 cells were treated with Ara-C in the presence or absence of Ferrostatin-1 (Fer-1), a potent inhibitor of ferroptosis. Flow cytometric analysis using Annexin V/PI staining demonstrated that Ara-C markedly increased the apoptotic rate of GC-1 cells. Notably, co-treatment with Fer-1 significantly attenuated this increase (Figure S4G), indicating that ferroptosis plays a critical role in Ara-C-induced germ cell toxicity. Consistently, biochemical assays showed that Ara-C treatment elevated malondialdehyde (MDA) levels and reduced glutathione (GSH) content, whereas Fer-1 partially reversed these changes (Figures 4G and S4F), further supporting the involvement of ferroptosis in this process.

Beyond ferroptosis, gene set scores analysis revealed marked suppression of DNA replication programs in the total SPG population. Subcluster examination indicated that this repression was confined to differentiated SPG, with undifferentiated SPG remaining largely unaffected (Figures 4H and 4I). At the gene level, replication factors such as *PCNA*, *Rnaseh1* and *Fen1* were significantly downregulated exclusively in differentiated SPG, whereas *Ssbp1* was repressed in both subtypes (Figure 4J). This selective inhibition of the DNA-replication machinery in differentiating spermatogonia suggests that Ara-C directly impairs their proliferative capacity, providing a mechanistic explanation for developmental arrest and SPG depletion. Oxidative-phosphorylation pathways were also consistently downregulated across both SPG clusters, further indicating widespread mitochondrial dysfunction (Figure S4H–S4J).

### The transcription factor Klf9 orchestrates a functional switch in Sertoli cells from niche support to stress survival

**Figure 5:**
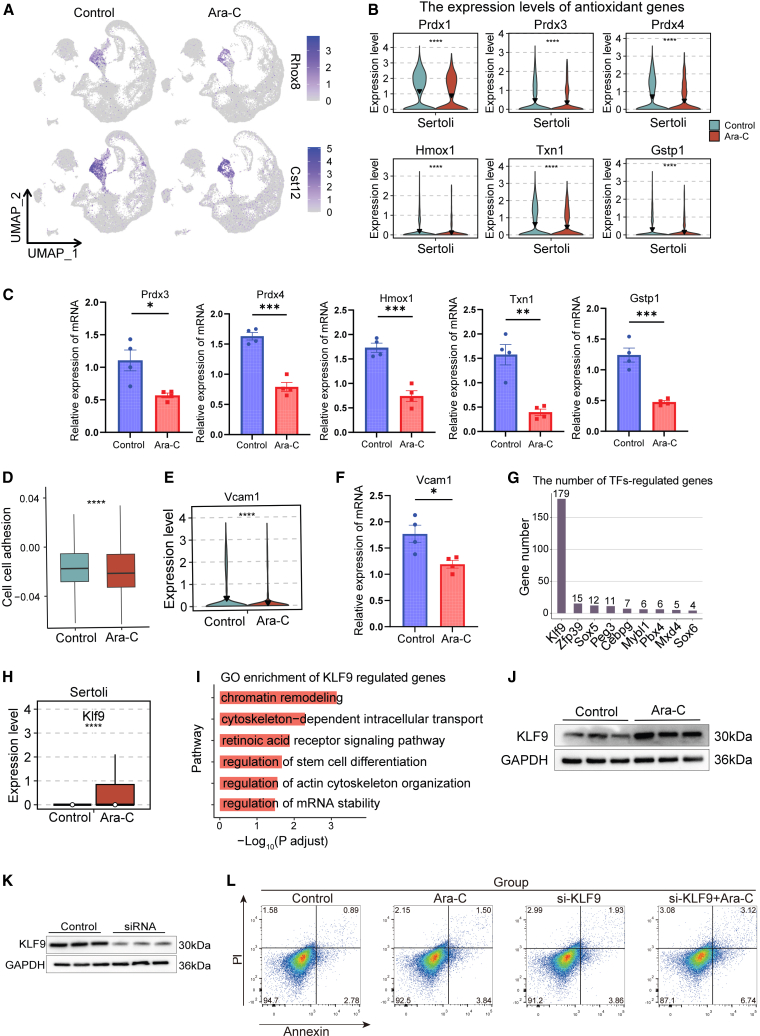
The transcription factor Klf9 orchestrates a functional switch in Sertoli cells from niche support to stress survival (A) UMAP plot showing the Sertoli cell cluster from control and Ara-C-treated (100 mg/kg b.w.) testes. Ara-C exposure caused a positional shift and internal redistribution, indicating large-scale remodeling of Sertoli cell transcriptional states. (B) Violin plot displaying upregulation of oxidative stress-related genes (*Prdx1*, *Prdx3*, *Prdx4*, *Txn1*, *Hmox1*, *Gstp1*) in Ara-C-treated Sertoli cells, revealing activation of antioxidant and detoxification programs. Statistical analysis by two-sided Wilcoxon rank-sum test: ∗*p* < 0.05; ∗∗*p* < 0.01; ∗∗∗*p* < 0.001; ∗∗∗∗*p* < 0.0001 vs. control. (C) qPCR validation confirming increased expression of *Hmox1*, *Prdx3*, *Prdx4*, *Txn1*, *and Gstp1*, consistent with the stress-adaptive transcriptional profile. Data are presented as mean ± SEM of *n* = 4 independent biological replicates. Statistical significance determined by Student’s *t* test: ∗*p* < 0.05; ∗∗*p* < 0.01; ∗∗∗*p* < 0.001; ∗∗∗∗*p* < 0.0001 vs. control. (D) and (E) Gene set score analysis showing strong suppression of cell adhesion-related gene programs in Ara-C-treated Sertoli cells. Statistical analysis by two-sided Wilcoxon rank-sum test: ∗*p* < 0.05; ∗∗*p* < 0.01; ∗∗∗*p* < 0.001; ∗∗∗∗*p* < 0.0001 vs. control. (F) qPCR analysis validating reduced expression of *Vcam1*, confirming weakened adhesion capacity and potential disruption of immune barrier integrity. Data are presented as mean ± SEM of *n* = 4 independent biological replicates. Statistical significance determined by Student’s *t* test: ∗*p* < 0.05; ∗∗*p* < 0.01; ∗∗∗*p* < 0.001; ∗∗∗∗*p* < 0.0001 vs. control. (G)–(I) Transcription factor (TF) regulatory network analysis identifying *Klf9*, *Zfp39*, and *Sox5* as major upstream regulators of Ara-C-associated DEGs. *Klf9* regulated the largest module (179 DEGs), which was enriched in pathways related to chromatin remodeling, stem cell differentiation, cytoskeletal organization, and cell adhesion. Statistical analysis by two-sided Wilcoxon rank-sum test: ∗*p* < 0.05; ∗∗*p* < 0.01; ∗∗∗*p* < 0.001; ∗∗∗∗*p* < 0.0001 vs. control. (J) Western blot confirming significant KLF9 upregulation in Ara-C-treated Sertoli cells. (K) Western blot analysis validating successful knockdown of KLF9 by siRNA. (L) Flow cytometric apoptosis analysis showing that KLF9 knockdown exacerbates Ara-C-induced apoptosis in Sertoli cells.

Following the observation that Ara-C treatment markedly reduced both the proportion and absolute number of Sertoli cells, we investigated the underlying mechanistic changes. UMAP visualization revealed a distinct Sertoli cell cluster that underwent a positional shift and altered internal distribution in treated samples, indicating a systemic remodeling of their transcriptional state (Figure 5A). This remodeling had critical functional consequences. The expression of key supportive markers was significantly impaired: *Rhox8*, a reproductive transcription factor essential for supporting sperm development, was strongly downregulated, pointing to a loss of germ cell support. Concurrently, *Cst12*, a secretory protein implicated in blood-testis barrier stability and antibacterial defense, was markedly reduced, suggesting a compromise in barrier function and local immunity. Paradoxically, alongside this loss of supportive function, Ara-C-exposed Sertoli cells mounted a robust oxidative stress response (Figure 5B). This included the marked downregulation of genes crucial for reactive oxygen species (ROS) clearance (*Prdx1, Prdx3, Prdx4*), cytoprotection (*Hmox1*), redox regulation (*Txn1*), and detoxification (*Gstp1*). qPCR independently confirmed this suppressed antioxidant profile, showing significantly reduced expression levels of *Hmox1, Prdx3, Txn1, Prdx4*, and *Gstp1* (Figure 5C). Rather than triggering a successful adaptive response, these findings indicate that Ara-C exposure severely impairs and overwhelms the intrinsic antioxidant defense system. This failure in ROS detoxification mechanisms provides a clear molecular basis for the unopposed oxidative stress, lipid peroxidation, and subsequent ferroptotic cell death observed in the germline.

Gene set scores analysis underscored a pronounced reduction in cell adhesion-associated gene programs in Ara-C-treated Sertoli cells (Figures 5D and 5E). This suppression was validated at the transcriptional level by qPCR, which showed significantly reduced *Vcam1* expression (Figure 5F). The coordinated downregulation of *Vcam1* and other adhesion molecules implies weakened Sertoli cell interactions with immune and endothelial cells, potentially loosening the immune barrier and impairing local immunoregulatory capacity. To uncover upstream regulatory drivers, transcription factor network analysis identified *Klf9*, *Zfp39* and *Sox5* as key regulators of Ara-C-associated DEGs, with *Klf9* controlling the largest module (179 DEGs) (Figures 5G–5I). These *Klf9*-regulated genes were enriched in chromatin remodeling, stem cell differentiation, and cytoskeleton organization, as well as cell adhesion and actin cytoskeleton regulation (Table S9). Single-cell analysis confirmed significant *Klf9* upregulation in Ara-C Sertoli cells, a change corroborated at the protein level by western blot (Figure 5J).

Functional assays demonstrated that knockdown of KLF9 exacerbated Ara-C-induced apoptosis in Sertoli cells (Figures 5K, 5L, and S5A), indicating that *Klf9* upregulation is an adaptive pro-survival response under oxidative stress; loss of *Klf9* removes this protection, leading to heightened cell death. TGFβ/BMP pathway activation in Ara-C Sertoli cells (Figure S5B). Key molecules such as *Tgfb3*, *Smad4* and *Bmpr2* were significantly upregulated, implicating pro-inflammatory and pro-fibrotic signaling in microenvironment remodeling. *Il31*, an inflammation-associated cytokine, likewise trended upward in expression. qPCR validated these transcriptional changes, fully consistent with single-cell data (Figure S5C). Together, these signals suggest that beyond oxidative stress and immune barrier disruption, Ara-C engages growth factor pathways that may influence Sertoli cell fate and extracellular matrix restructuring.

### Ara-C triggers testosterone suppression and inflammatory activation in Leydig cells

Single-cell transcriptomic profiling revealed a mixed-pattern alteration in steroidogenic enzyme expression within Ara-C-treated Leydig cells (Figure S5D). We observed marked downregulation of key rate-limiting and terminal enzymes for testosterone synthesis, including *Cyp11a1*, *Hsd17b3* and *Hsd17b12*. Conversely, genes encoding intermediate cholesterol metabolism enzymes, such as *Hsd17b7*, *Hsd3b6* and *Srd5a1*, were significantly upregulated. Given the strong Leydig cell specificity of these enzymes, this bidirectional response suggests a compensatory upregulation of specific metabolic steps within an otherwise impaired steroidogenic network (Figure S5E). Nonetheless, the loss of critical biosynthetic enzymes is predicted to constrain overall testosterone production.

Pathway enrichment analyses (Figure S5F) showed activation of *HIF-1* signaling, protein processing in the ER, and *FoxO* signaling, alongside suppression of glutathione metabolism and drug metabolism, suggestive of oxidative stress and impaired detoxification capacity (Table S10). Heatmaps revealed strong induction of hypoxia-associated genes (*Hif1a*, *Egln1*, *Arnt*, *Ldha*, *Vegfa*), supported by elevated hypoxia, inflammatory response, and cytokine gene set scores in Ara-C Leydig cells (Figure S5G). These findings indicate that Ara-C may indirectly induce a hypoxic phenotype via altered blood flow, metabolic disturbances, or oxidative stress. The inflammatory response and hypoxia score was markedly increased (Figure S5H), pointing to an enhanced immunoregulatory role of Leydig cells under stress. Correspondingly, cytokine profiling (Figure S5I) revealed significant upregulation of multiple chemokines (*Cxcl1*, *Cxcl10*, *Cxcl12*, *Cxcl16*), immune receptors (*Ifngr1*, *Il6st*), and growth factors (*Vegfd*, *Pdgfra*). Notably, *Il31*, which was also elevated in Sertoli cells, showed pronounced activation. Functionally, these changes suggest that in the damaged environment, Leydig cells not only sustain endocrine functions but also engage in paracrine immunomodulation, potentially facilitating immune cell recruitment and contributing to local testicular inflammation.

### Ara-C remodels the testicular cellular communication network

**Figure 6:**
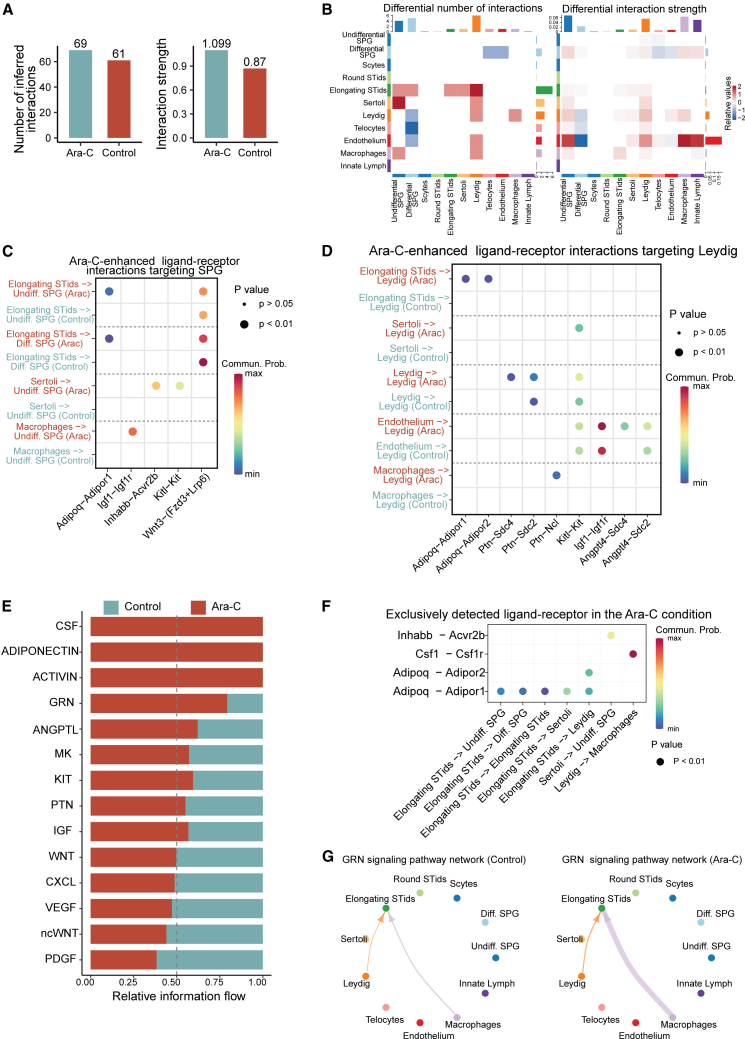
Ara-C remodels the testicular cellular communication network (A) Bar plot comparing the total number and interaction strength of predicted ligand-receptor pairs between control and Ara-C-treated testes. (B) Heatmap summarizing cell type-specific changes in the number and strength of interactions. (C) and (D) Dot plots displaying key ligand-receptor interactions that were significantly strengthened by Ara-C exposure, targeting (C) undifferentiated/differentiated spermatogonia (SPG) and (D) Leydig cells. Dot color represents the interaction strength, and dot size indicates the statistical significance level (-log_10_ (*p* value)). (E) Bar plot of RankNet analysis depicting differential information flow across major signaling pathways. Bars represent the relative signaling activity in control (blue) and Ara-C-treated (red) conditions. (F) Bubble plot visualizing ligand-receptor pairs (e.g., *Inhabb*-*Acvr2b*, *Adipoq*-*Adipor1*/*2*, *Csf1*-*Csf1r*) that were specifically identified as active in the Ara-C condition but not in the control. (G) Network plot of the glucagon related neuropeptide (GRN) signaling pathway under Ara-C treatment, illustrating denser and stronger intercellular connections, particularly from macrophages to elongating spermatids (elongating STids).

To investigate how Ara-C alters intercellular communication, we applied CellChat to single-cell transcriptomes from control and treated testes. Ara-C treatment robustly enhanced global cell-cell communication, increasing both the number (69 vs. 61) and strength (1.099 vs. 0.87) of interactions, suggesting a hyperactive microenvironment indicative of a stress response (Figure 6A). As illustrated in Figure 6B, analysis of the overall interaction intensity revealed profound Ara-C-induced perturbations within the testicular microenvironment. Notably, the most dramatic alterations in cell-cell communication were observed between Sertoli cells and undifferentiated spermatogonia, as well as between Leydig cells and elongating spermatids. The pronounced signaling shift between Sertoli cells and undifferentiated spermatogonia strongly implies a disruption of the somatic niche, which likely contributes to the aforementioned imbalance in spermatogonial self-renewal and stress-induced differentiation. Concurrently, the altered interaction intensity between Leydig cells and elongating spermatids indicates an impairment in the vital endocrine/paracrine support required for post-meiotic germ cell maturation.

Similarly, macrophages gained strength without increasing interaction number, while differentiated SPG demonstrated the largest decrease (Figures S6A and S6B). Together, these findings highlight a divergence in cell-type-specific communication responses to Ara-C, with immune cells such as macrophages maintaining intensified signaling, whereas differentiated spermatogonia exhibit pronounced communication attenuation.

We next identified the ligand-receptor pairs driving this enhanced connectivity. For undifferentiated SPG, strengthened interactions included *Adipoq*-*Adipor1* and *Wnt3*-(*Fzd3*+*Lrp6*) from spermatids, *Kitl*-*Kit* and *Inhabb*-*Acvr2b* from Sertoli cells, and *Igf1*-*Igf1r* from macrophages (Figure 6C). For Leydig cells, key augmented interactions included paracrine signals (*Adipoq*-*Adipor1/2* from spermatids; *Kitl*-*Kit* from Sertoli; *Ptn*-*Ncl/Sdc2/4* from endothelium; *Igf1*-*Igf1r* from macrophages) and autocrine loops (*Angptl4*-*Sdc2*/*4*, *Kitl*-*Kit*) (Figure 6D).

RankNet analysis revealed that ligand-receptor pairs associated with the platelet-derived growth factor (PDGF), noncanonical wingless-related integration site (WNT) (ncWNT), and vascular endothelial growth factor (VEGF) signaling pathways showed reduced overall information flow following Ara-C treatment. In contrast, ligand-receptor pairs within the ACTIVIN, ADIPONECTIN, and colony-stimulating factor (CSF) pathways formed newly induced signaling networks specific to the Ara-C group (Figure 6E). Bubble plot visualization further identified the corresponding interactions as *Inhabb*-*Acvr2b*, *Adipoq*-*Adipor1*/*2*, and *Csf1*-*Csf1r*, which were detected only under Ara-C exposure (Figure 6F). Moreover, although glucagon related neuropeptide (GRN) signaling was not prominent in the initial screens, it became markedly elevated, suggesting an underappreciated role for granulin-mediated intercellular communication. Visualization of pathway networks showed that GRN signaling under Ara-C formed denser and stronger connections, particularly from macrophages to elongating STids (Figure 6G). By contrast, the ncWNT network displayed reduced connectivity from Leydig cells to SPG (Figure S6C), indicating selective inhibition of WNT-mediated crosstalk.

## Discussion

Chemotherapeutic improvement has profoundly increased survival among pediatric and adolescent cancer patients, but the accompanying rise in treatment-related late effects has created a parallel need to safeguard long-term quality of life.^8^^,^^10^ Among these sequelae, chemotherapy-induced gonadotoxicity poses a particularly serious challenge for prepubertal boys, who cannot rely on sperm cryopreservation to preserve fertility.^9^^,^^31^ Although cytarabine (Ara-C) remains indispensable for acute leukemia therapy, its testicular toxicity has long been recognized without mechanistic clarity.^26^

In this study, we integrated bulk and single-cell RNA-sequencing with metabolomics in a prepubertal murine model to provide a cell-resolved resolution of how Ara-C perturbs the testicular ecosystem. This model was specifically selected to capture a clinically critical developmental window, as the prepubertal testis is highly susceptible to chemotherapeutic injury with long-term implications for future fertility. By evaluating Ara-C as a single agent rather than within combination regimens, we isolated its intrinsic gonadotoxic potential, thereby minimizing confounding variables from other cytotoxic drugs. To ensure translational relevance, we employed a dose-dependent administration strategy (10–100 mg/kg) based on human equivalent dose (HED) conversion using the U.S. Food and Drug Administration (FDA)-recommended body surface area (BSA) normalization method^32^ (conversion factor ≈3). Under this framework, the low dose (10 mg/kg) corresponds to approximately 30 mg/m^2^ in humans, mirroring pediatric maintenance therapy regimens. Conversely, the high dose (100 mg/kg) approximates 300 mg/m^2^, which falls within the range of standard therapeutic induction or consolidation doses while remaining below high-dose Ara-C (HiDAC) protocols.^33^ Consequently, this experimental design effectively models a clinical exposure spectrum spanning from maintenance-level to standard chemotherapy intensity, providing a controlled framework to delineate toxicity thresholds and mechanistic targets relevant for fertility preservation in pediatric cancer survivors.

Consistent with clinical reports of long-term hypogonadism in Ara-C-treated survivors, our *in vivo* experiments revealed a clear dose-dependent pattern of spermatogenic failure, seminiferous-tubule disorganization, and hormonal suppression. The concomitant decline in testosterone and luteinizing hormone, together with Leydig-cell transcriptional repression of *Cyp11a1* and *Hsd17b3*, indicates combined primary and secondary hypogonadism.^34^ Single-cell profiling extended these findings by showing that virtually every somatic and germline compartment was perturbed, collectively contributing to testicular dysfunction rather than resulting from isolated germ-cell depletion alone.

Among germline lineages, SPG and elongating spermatids emerged as the most vulnerable. Differential expression and pathway enrichment analyses consistently revealed opposite transcriptional directions in these two populations: global downregulation and cell-cycle arrest in SPG, in contrast to broad metabolic activation and ER stress induction in elongating spermatids. Such polarity highlights the stage-specific sensitivities of mitotic versus post-meiotic germ cells. A key mechanistic insight is that Ara-C provokes ferroptosis, a form of non-apoptotic cell death driven by lipid peroxidation, across multiple germline populations. The convergence of glycolysis enhancement, glutathione pathway engagement, and oxidative phosphorylation repression predicted oxidative stress; subsequent transcriptomic, gene set, and biochemical validation confirmed ferroptotic activation through increased levels of MDA, a lipid peroxidation product, and decreased levels of reduced GSH. Given that ferroptosis depends on iron-catalyzed ROS propagation,^35^ these data suggest that disruption of redox homeostasis is a proximal driver of Ara-C-induced germ cell death.

In parallel, DNA replication and repair programs were markedly suppressed, especially within differentiated SPG. Specific downregulation of replication factors (*PCNA, Rnaseh1, Fen1*) together with persistence of unrepaired double-strand break signatures implies that Ara-C exerts a direct cytotoxic effect through incorporation into replicating DNA and blockade of elongation—a canonical pharmacodynamic mechanism of Ara-C^23^—now demonstrated here at single-cell resolution. This selective replication arrest explains the pronounced loss of differentiating SPG and aligns with the acute depletion phenotype observed histologically. Furthermore, beyond these cytotoxic and proliferation-inhibitory effects, the relative accumulation of primary spermatocytes and spermatids observed in our data suggests an additional, complementary mechanism. It is highly plausible that the genotoxic stress induced by Ara-C disrupts the precise balance between spermatogonial self-renewal and differentiation. Specifically, Ara-C may prematurely drive SPG out of their undifferentiated state, promoting excessive differentiation and inappropriately accelerating the mitosis-to-meiosis transition. Such a stress-induced response would synergistically exacerbate the exhaustion of the spermatogonial pool while transiently causing the downstream overaccumulation of meiotic and post-meiotic cells. Thus, Ara C compromises germline integrity through a multifaceted attack: oxidative lipid peroxidation leading to ferroptosis, nucleoside analog-mediated interference with replication dynamics, and potentially, stress-induced premature differentiation.

Sertoli cells, which orchestrate spermatogenesis and maintain the blood-testis barrier, exhibited both depletion and profound transcriptional reprogramming.^36^ Downregulation of support genes (*Rhox8*, *Cst12*) and adhesion molecules (*Vcam1*) suggests weakening of the structural and immunological niche.^37^^,^^38^ Simultaneous activation of antioxidant genes (*Hmox1*, *Prdx3/4*, *Txn1*) and the stress-responsive transcription factor *Klf9* defines an adaptive response aimed at limiting ROS damage.^39^^,^^40^ Functional assays showing increased apoptosis upon *Klf9* knockdown indicate that *Klf9* upregulation confers partial protection. Yet, despite this adaptation, persistent TGFβ/BMP pathway activation implies fibrotic remodeling and inflammation within the Sertoli compartment, potentially leading to sustained long-term microenvironmental dysfunction. Such long-term microenvironmental dysfunction may therefore also impair the proliferation and differentiation of germ cells, in addition to the direct deleterious effects of cytarabine.^41^ These findings expand the concept of Sertoli-cell plasticity under toxic stress: while attempting survival via transcriptional reprogramming, they lose their fundamental support roles, thereby amplifying germ-cell vulnerability.

Our data further reveal that Leydig cells, traditionally viewed solely as endocrine components,^42^ undergo complex stress-related remodeling. The concomitant downregulation of terminal steroidogenic enzymes and upregulation of intermediate cholesterol pathways indicate a maladaptive, energy-inefficient attempt to sustain androgen output. Enrichment of HIF-1 and *FoxO* signaling and induction of hypoxia-related genes (*Hif1a*, *Vegfa*, *Egln1*) reflect metabolic and oxygen-sensing perturbations.^43^ The strong activation of inflammatory chemokines (*Cxcl1*, *Cxcl10*, *Cxcl12*)^44^ and cytokines (*Il31*, *Ifngr1*)^45^ suggests that Ara-C converts Leydig cells into a paracrine inflammatory hub. Together with similar *Il31* upregulation in Sertoli cells, these observations depict a coordinated inflammatory crosstalk that may trigger broader immune-cell recruitment, altering the immune-privileged testicular milieu and perpetuating tissue injury.

The testicular niche responds as a network. Cell-cell communication analysis reveals a hyperconnected microenvironment centered on Sertoli hubs, with induction of ACTIVIN, ADIPONECTIN, CSF, and GRN pathways and reduced information flow through PDGF, noncanonical WNT, and VEGF. Although such hyperconnectivity is likely an adaptive attempt to stabilize homeostasis, the specific pathway shifts we observe are compatible with reduced progenitor support and persistent inflammatory tone. Methodologically, communication inferences can be influenced by cell composition and library size, so down-sampling or replication-aware analyses will further strengthen these network conclusions.^46^ Even with this caveat, the convergence of single-cell transcriptional states and ligand-receptor topology points to actionable nodes at the level of adhesion, growth factor signaling, and metabolic stress responses.

A notable finding is the persistence of discrete transcriptomic and metabolic alterations in unexposed F1 offspring. The enrichment of inflammatory and nucleotide and energy pathways suggests transmission of a stress imprint^47^ at least one generation. We use the term intergenerational to reflect changes in F1 without direct exposure. Defining the mediators of this imprinting, including small RNAs and DNA methylation in sperm,^48^^,^^49^ and testing whether these effects extend to F2,^50^ will be essential to delineate scope and mechanism and to understand human relevance.

These insights have immediate translational implications. The combined evidence for ferroptosis and replication arrest prioritizes two orthogonal classes of interventions. First, redox and lipid-peroxide-focused strategies,^51^ such as iron chelation or lipid peroxide scavenging, merit testing to blunt early germline loss. Second, approaches that preserve replication competence in differentiating spermatogonia may prevent arrest-induced depletion. Because Sertoli cells shift toward *Klf9*-mediated stress survival while losing adhesion and trophic outputs, targeted modulation of *Klf9*^52^ and of TGFβ/BMP^53^ and adhesion programs could restore niche function. Notably, the stress-induced upregulation of *Klf9* aligns with its conserved role as an adaptive transcriptional regulator,^54^ extending this paradigm to the chemotherapy-injured testis and reinforcing its plausibility as a survival factor. While we established a coherent mechanistic link between *Klf9* activation and Sertoli cell survival via *in vivo* network inference and functional perturbation, we acknowledge that cell-type-specific genetic models represent the definitive standard. Future studies employing conditional knockout or overexpression strategies will be crucial to further delineate the precise cell-autonomous contributions of *Klf9* to testicular homeostasis and injury responses.

In Leydig cells, dampening inflammatory rewiring while maintaining steroidogenesis may improve the endocrine milieu during and after chemotherapy.^55^ All candidate interventions should be screened for the ability to preserve fertility without compromising antileukemic efficacy.^56^ This requirement motivates evaluation in leukemia-bearing models, attention to pharmacokinetics and tissue distribution, and careful sequencing relative to Ara-C dosing, to better approximate clinically relevant conditions in human patients.

### Limitations of the study

Our study has limitations. First, while our study employed a dose-dependent strategy, the use of a fixed 4-week treatment window in a murine model inherently constrains both dose-time mapping and species generalizability. Specifically, this time frame primarily captures early-to-subacute cellular responses, leaving the long-term reversibility of Ara-C-induced injury and potential compensatory mechanisms, particularly in somatic cells, to be fully elucidated. Future studies incorporating multi-dose recovery designs that span acute to chronic phases, alongside cross-species validation, will be essential to refine thresholds for reversible versus irreversible damage and to determine whether testicular repair processes attenuate cytarabine-induced effects over time.

Second, inherent interspecies differences mandate caution when extrapolating murine findings to clinical settings. Pharmacokinetically, rodents typically exhibit faster metabolic clearance of Ara-C compared to humans, which may influence absolute systemic exposure and tissue accumulation despite BSA normalization.^57^ Biologically, the temporal dynamics of testicular development and spermatogenesis differ significantly across species; the 4-week treatment covers a substantial portion of the murine prepubertal window, whereas the corresponding developmental phase in humans is more protracted.^58^ Consequently, while our model elucidates key mechanistic pathways, future investigations employing cross-species validation and human cohort analyses are warranted to refine the translational applicability of these findings.

Meanwhile, although single-cell profiling offers high-resolution insights into cellular states and intercellular communication, changes in cell abundance,^59^ particularly the depletion of specific populations such as SPG, can reduce statistical power to detect significant ligand-receptor interactions and increase variability in average expression estimates, thereby affecting both the number and inferred strength of predicted interactions. Therefore, alterations in inferred communication networks likely reflect a combination of genuine modulation of signaling activity and shifts in cellular composition. In this study, we focused on relative, network-level communication trends across conditions rather than absolute interaction counts for individual sender-receiver pairs,^60^ thereby minimizing overinterpretation of abundance-driven effects. Future studies incorporating balanced sampling strategies, orthogonal approaches for cell-cell signaling inference, and spatial transcriptomics will further enhance robustness and enable more precise localization of injury-associated signaling gradients within the tissue microenvironment.^61^

Beyond these analytical insights, while our study reveals severe Ara-C-induced impairment of prepubertal spermatogenesis, we did not perform *in vivo* mating experiments. Consequently, future long-term breeding assays are needed to definitively quantify the functional consequences on F0 fertility, particularly in terms of pregnancy rates and litter sizes, as well as to comprehensively assess the intergenerational impacts on the reproductive health of F1 and subsequent generations. Finally, although our biochemical and transcriptional data are consistent with ferroptosis and replication arrest, causal links will require *in vivo* rescue experiments using ferroptosis inhibitors or iron chelators, and genetic or pharmacologic modulation of replication programs, together with readouts of long-term fertility.

In sum, by integrating multi-omics across germ and somatic compartments in the prepubertal testis, we outline a mechanistic framework in which Ara-C compromises spermatogenesis through redox imbalance with ferroptosis and replication blockade, provokes stress-adaptive but support-limiting reprogramming of Sertoli cells, and converts Leydig cells into an inflammatory and hypoxia-associated state, all within a niche that becomes hyperconnected yet functionally maladaptive. The persistence of molecular alterations in F1 offspring indicates intergenerational imprinting of this stress response. This framework is designed to identify vulnerable lineages and actionable pathways that can inform fertility-preserving interventions without compromising antileukemic efficacy and it prioritizes ferroptosis, ER stress and lipid handling, the Klf9 and TGFβ axis in Sertoli cells, and inflammatory rewiring in Leydig cells as entry points for rational therapy development.

## Resource availability

### Lead contact

Further information and requests for resources and reagents should be directed to and will be fulfilled by the Lead Contact and corresponding author, Yali Song (syl@smu.edu.cn).

### Materials availability

This study did not generate new unique reagents.

### Data and code availability

• The datasets generated in this study have been deposited at the National Genomics Data Center (NGDC), China National Center for Bioinformation/Beijing Institute of Genomics, Chinese Academy of Sciences, under BioProject accession number PRJCA058617 (https://ngdc.cncb.ac.cn/bioproject/browse/PRJCA058617).
• The raw sequencing data have been deposited in the Genome Sequence Archive (GSA) under accession numbers CRA039901, containing raw FASTQ files for bulk RNA sequencing from F0 Ara-C-treated testes and untreated F1 offspring, and CRA039929, containing raw FASTQ files for single-cell RNA sequencing from control and Ara-C-treated testes (https://ngdc.cncb.ac.cn/gsa).
• Processed matrix and result files are available in the OMIX database under the same BioProject: OMIX015234 for processed bulk RNA-seq expression matrices and associated result files (https://ngdc.cncb.ac.cn/omix/release/OMIX015234), OMIX015233 for processed single-cell RNA-seq expression matrices and associated result files (https://ngdc.cncb.ac.cn/omix/release/OMIX015233), and OMIX015235 for the untargeted metabolomics processed dataset and associated result files (https://ngdc.cncb.ac.cn/omix/release/OMIX015235).
• All other data supporting the findings of this paper are available within the article and supplemental information. Additional data are available from the lead contact, Yali Song (syl@smu.edu.cn), upon reasonable request. The analysis code used in this study is archived on Zenodo (https://doi.org/10.5281/zenodo.21397301) and is also available at https://github.com/syx6/Ara-C.

## Acknowledgments

The authors gratefully acknowledge financial support from the 10.13039/501100021171Guangdong Basic and Applied Basic Research Foundation (grant nos. 2025A1515012333 and 2026A1515012638); Dongguan Basic Research Foundation (grant nos. 20251800400372, 20251800400382); Funded Project of Clinical Research Fund of Guangdong Medical Association—Livzon Special Program (grant no. 2024LZ-A1002).

## Author contributions

S.X., H.L., S.H., and Y.S.: conceived and designed the study; S.X., J.W., J.D., W.Y., Y.Sun, X.Y., X.C., J.C., and H.W.: performed experiments, data collection, and/or data curation; J.W., J.D., Y.Sun, X.Y., X.C., J.C., and H.W.: performed formal analyses and contributed to methodology or software; S.X., J.W., X.Y., and S.H.: prepared visualizations; S.X.: wrote the original draft; H.L. and Y.S.: supervised the study, acquired funding, and reviewed and edited the manuscript; all authors read and approved the final manuscript.

## Declaration of interests

The authors declare no competing interests.

## STAR★Methods

### Key resources table

**Table:** 

REAGENT or RESOURCE	SOURCE	IDENTIFIER
**Antibodies**		

KLF9 antibody	Abmart	Cat# MG418446
GAPDH antibody	Cell Signaling Technology	Cat# 97166S; RRID: AB_2756824

**Chemicals, peptides, and recombinant proteins**		

Cytarabine (Ara-C)	Commercial source	N/A
Ferrostatin-1	MCE	Cat# HY-100579
Collagenase IV	Sigma-Aldrich	1 mg/mL
DNase I	Thermo Fisher	100 U/mL

**Critical commercial assays**		

Mouse Testosterone ELISA Kit	COIBO BIO	Cat# CB10466-Mu
Mouse LH ELISA Kit	COIBO BIO	Cat# CB10542-Mu
Annexin V-FITC/PI Apoptosis Detection Kit	Elabscience	Cat# E-CK-A211
Total Glutathione Assay Kit	Beyotime Biotechnology	Cat# S0053
Lipid Peroxidation MDA Assay Kit	Beyotime Biotechnology	Cat# S0131

**Experimental models: Cell lines**		

15P-1 mouse Sertoli cell line	ATCC	N/A
GC-1 SPG mouse spermatogonial cell line	Pricella	Cat# CL-0600

**Experimental models: Organisms/strains**		

C57BL/6 male mice, 2 weeks old	Animal Center of Shenzhen People’s Hospital	Ethics approval AUP-220302-XSY-006-01

**Deposited data**		

Bulk RNA-seq raw data	NGDC Genome Sequence Archive	NGDC Genome Sequence Archive: CRA039901; BioProject: PRJCA058617
Single-cell RNA-seq raw data	NGDC Genome Sequence Archive	NGDC Genome Sequence Archive: CRA039929; BioProject: PRJCA058617
Processed bulk RNA-seq data	NGDC OMIX	NGDC OMIX: OMIX015234
Processed single-cell RNA-seq data	NGDC OMIX	NGDC OMIX: OMIX015233
Processed metabolomics data	NGDC OMIX	NGDC OMIX: OMIX015235
Analysis code	This paper	Zenodo: https://doi.org/10.5281/zenodo.21397301; GitHub: https://github.com/syx6/Ara-C

**Software and algorithms**		

Cell Ranger	10X Genomics	v7.1
Seurat	Butler et al.^62^	v4.1.2/v5
DoubletFinder	McGinnis et al.^63^	https://github.com/chris-mcginnis-ucsf/DoubletFinder
Slingshot	Street et al.^64^	N/A
SCENIC	Aibar et al.^65^	v1.1.2
clusterProfiler	Yu et al.^66^	v4.1.0
CellChat	Jin et al.^67^	v1.1.3
R	R Foundation	v4.1.2

**Other**		

Analysis code	This paper	https://doi.org/10.5281/zenodo.21397301; https://github.com/syx6/Ara-C

### Experimental model and study participant details

#### Animal model and treatment

Male C57BL/6 mice at postnatal age 2 weeks (prepubertal developmental stage) were housed under specific pathogen-free (SPF) conditions in a controlled environment (22°C, 50% humidity, 12-h light/dark cycle) and randomly assigned to treatment groups. All animal subjects used in this study were male, consistent with the focus on prepubertal testicular toxicity. The experimental group received daily intraperitoneal injections of cytarabine (0, 10, 50, or 100 mg/kg b.w. in PBS, dissolved in 0.2 mL PBS), whereas the control group was administered an equal volume of PBS (0.2 mL) on the same schedule. The treatment regimen was maintained for 4 weeks. Twenty-four hours after the final injection, all mice were anesthetized with isoflurane and euthanized by cervical dislocation. Following euthanasia, the testes were carefully dissected. One testis from each animal was fixed in 4% paraformaldehyde for 24 h at 4°C for subsequent histopathological examination. The contralateral testis from a subset of mice was immediately processed for downstream multi-omics analyses, including single-cell RNA sequencing and transcriptomics. All animal experiments were approved by the Animal Care and Use Committee of Animal Center of Shenzhen People’s Hospital (ethical approval number: AUP-220302-XSY-006-01) and were conducted in accordance with institutional guidelines and relevant regulatory standards for animal research.

#### Animal grouping

A total of 20 male prepubertal mice (2-week-old) were randomly assigned to either the Control (*n* = 10) or the 100 mg/kg Ara-C treatment group (*n* = 10). Given the extreme fragility of pre-weaning pups subjected to continuous intraperitoneal injections for one month, combined with the chemotherapeutic toxicity of Ara-C, 3 mice in the Ara-C group were lost during the experimental period. This resulted in a final available sample size of *n* = 10 for Controls and *n* = 7 for the Ara-C group. Tissues from surviving animals were allocated based on specific assay requirements. Due to the high consistency of normal baseline parameters, a statistically sufficient subset of control mice was used for certain functional evaluations (e.g., *n* = 6 for sperm count), whereas all surviving Ara-C mice (*n* = 7) were analyzed to account for individual variability in drug-induced toxicity. The specific *n* number for each assay is explicitly detailed in the respective figure legends.

### Method details

#### Hematoxylin–eosin (H&E) staining

The collected tissues were fixed in 4% paraformaldehyde, followed by dehydration in a series of ethanol dilutions and embedding in paraffin. Paraffin blocks were then sectioned into 4 μm thickness and mounted on glass slides. Hematoxylin and eosin (H&E) staining were performed on the sections. Images were observed and captured by an imaging microscope.

#### Serum collection and hormone assay

Blood samples were collected from Ara-C-treated mice (*n* = 7) and control mice (*n* = 10). After clotting at room temperature for 30 min, the blood was centrifuged at 3,000 × g for 15 min at 4°C to obtain serum. The serum was aliquoted and stored at −80°C until analysis. Serum testosterone concentration was measured using a Mouse Testosterone ELISA Kit (COIBO BIO, Cat.# CB10466-Mu), and luteinizing hormone (LH) concentration was determined using a Mouse LH ELISA Kit (COIBO BIO, Cat.# CB10542-Mu). All procedures were performed strictly according to the respective manufacturer’s instructions. All samples were assayed in duplicate, and the absorbance was measured using a microplate reader.

#### Sperm collection and counting

After the experimental period, the cauda epididymides were carefully dissected from each mouse, with sample sizes of *n* = 7 for the Ara-C-treated group and *n* = 6 for the control group. Spermatozoa were released by puncturing the epididymal tubules and incubated in 1 mL of pre-warmed phosphate-buffered saline (PBS) at 37°C for 15 min to allow for sperm dispersion. The sperm suspension was then gently diluted at a ratio of 1:100 in PBS. Sperm counting was performed using a standard hemocytometer (Neubauer chamber) under a light microscope. The number of spermatozoa from at least two independent counts per sample was averaged, and the data were expressed as sperm count per cauda epididymis.

#### RNA isolation and quantitative real-time PCR (qRT-PCR)

Total RNA was isolated from the mouse Sertoli cell line 15P-1 using the FastPure Cell/Tissue Total RNA Isolation Kit V2 (Vazyme), according to the manufacturer’s instructions. Subsequently, 1 μg of total RNA was reverse-transcribed into cDNA using the HiScript IV ALL-in-One Ultra RT SuperMix for qPCR (Vazyme). Quantitative PCR (qPCR) was performed in a 20 μL reaction volume using the ChamQ Universal SYBR qPCR Master Mix (Vazyme) on a QuantStudio 6 Flex System (Thermo Fisher Scientific). The thermal cycling conditions were as follows: initial denaturation at 95°C for 30 s, followed by 40 cycles of 95°C for 30 s and 60°C for 10 s. Amplification of target genes was carried out using the gene-specific primers (Table S11). The relative mRNA expression levels of target genes were normalized to the endogenous control *Gapdh* and calculated using the comparative 2^∧^-ΔΔCt method.

#### Cell culture and transfection

The 15P-1 mouse Sertoli cell line was obtained from the American Type Culture Collection (ATCC). Cells were cultured in Dulbecco’s Modified Eagle Medium (DMEM) supplemented with 10% fetal bovine serum (FBS; Gibco), 100 U/mL penicillin, and 100 μg/mL streptomycin, and maintained at 37°C in a humidified 5% CO_2_ atmosphere. The cell line was regularly tested and confirmed to be mycoplasma-free, and experiments were performed using cells within 20 passages. For all procedures, cells were treated or transfected when reaching 50–80% confluency. Separately, the GC-1 SPG cell line (Pricella, CL-0600) was propagated under identical culture conditions using DMEM with 10% FBS and 1% penicillin-streptomycin. The 15P-1 Sertoli cell line represents a male gonadal somatic cell lineage; GC-1 SPG cells represent a male spermatogonial germ-cell lineage. Sex as a biological variable is therefore intrinsic to the experimental model and was not analyzed as an independent variable.

#### Cell viability assays

Cell viability was evaluated using the CCK-8 kit (TargetMol, Cat#:C0005) according to the manufacturer’s instructions. 8.0×10^3^ 15P-1 cells (cell density of 8.0 × 10^5^ cells/mL) were seeded into a 96-well plate and treated with Cytarabine. Briefly, the CCK-8 solution (10 μL/well) was added to cells in each well with 100 μL of medium and further incubated at 37 °C for 1–4h. The plates were measured at the absorbance of 450 nm and cell viability was determined for each group via comparison to the untreated control. A microplate reader (BioTeck, USA) was used to determine OD value at 450 nm.

#### siRNA-mediated knockdown of KLF9

To knock down KLF9 expression, 15P-1 cells were seeded in 6-well plates at a density of 2×10^5^ cells per well and cultured until they reached 60–70% confluence. Transfection was performed using the siRNA-mate plus transfection reagent (GenePharma, Cat# G04027) according to the manufacturer’s instructions. The KLF9-specific siRNA sequences (synthesized by GenePharma, Jiangsu, China) were as follows: Sense (S): 5′-GGAGUGACCAUCUCACCAATT-3′; Antisense (AS): 5′-UUGGUGAGAUGGUCACUCCTT-3′. Cells transfected with a non-targeting scrambled siRNA were used as the negative control. The cells were harvested 48 to 72 h post-transfection for subsequent efficiency validation and functional analyses.

#### Western blot

Cell pellets were lysed with cold RIPA buffer (Thermo Scientific), sonicated, and incubated on ice for 20 min followed by centrifugation at 12,000 rpm at 4°C for 15 min. Protein quantification was performed using a Pierce BCA Protein Assay kit (Thermo Scientific). Antibodies for Western blotting included KLF9 (Abmart, Cat#:MG418446), and GAPDH (Cell Signaling Technology, Cat #: 97166S).

#### Flow cytometric analysis of apoptosis

Apoptosis was quantified by flow cytometry using an Annexin V-FITC/PI dual-staining assay. For 15P-1 cells, seeded overnight in 12-well plates (5.0 × 10^5^ cells/well), treatments consisted of Ara-C at the IC_50_ concentration or respective siRNA for 24 h. Following treatment, cells were harvested and stained per the manufacturer’s protocol for the Annexin V-FITC/PI Apoptosis Detection Kit (Elabscience, #E-CK-A211). In parallel, GC-1 SPG cells (Pricella, #CL-0600), maintained in DMEM supplemented with 10% FBS and 1% penicillin-streptomycin at 37°C with 5% CO_2_, were subjected to rescue experiments. These cells were treated for 24 h with Ara-C (IC_50_) either alone or in combination with 0.5 μM Ferrostatin-1 (MCE, #HY-100579). Post-treatment, GC-1 cells were similarly processed for Annexin V-FITC/PI staining. Apoptotic rates for both cell lines were subsequently determined by flow cytometry. All experiments were conducted with three independent biological replicates.

#### Measurement of malondialdehyde (MDA) and glutathione (GSH) levels

Cells were seeded in six-well plates and treated under conditions consistent with the IC_50_ assay. Intracellular glutathione (GSH) levels were measured using a Total Glutathione Assay Kit (S0053, Beyotime Biotechnology) according to the manufacturer’s instructions. Briefly, cells were washed with 1× PBS, harvested, and resuspended in three volumes of Protein Removal Reagent M. Cell lysis was achieved by two rapid freeze–thaw cycles (liquid nitrogen and 37 °C water bath). Appropriate detection reagents were added, samples were incubated for 25 min, and absorbance was measured at 412 nm using a microplate reader. GSH concentrations were calculated based on a standard curve.

Malondialdehyde (MDA) levels were determined using a Lipid Peroxidation MDA Assay Kit (S0131, Beyotime Biotechnology) following the manufacturer’s protocol. Cells were washed with 1× PBS, collected, and resuspended in 100 μL PBS. Protein concentrations were measured using a BCA Protein Assay Kit. Detection reagents were added, and samples were heated at 100 °C for 15 min. Absorbance was recorded at 532 nm using a microplate reader. MDA levels were normalized to total protein content.

#### Testicular single-cell suspension preparation

Tissue Dissociation: Minced testicular tissues were enzymatically digested in 2 mL of digestion buffer containing Collagenase IV (1 mg/mL, Sigma-Aldrich) and DNase I (100 U/mL, Thermo Fisher) at 37°C for 30 min with gentle agitation (150 rpm). Seminiferous tubules were dissociated by pipetting 20 times with a 1 mL wide-bore tip. The reaction was quenched with 10% FBS in DMEM. Cell Filtration and Washing: Cell suspensions were filtered through a 40-μm cell strainer, centrifuged at 300 × g for 5 min, and resuspended in PBS +0.04% BSA. Viability and Count: Cell viability (>90%) was confirmed by Trypan Blue exclusion (Countess II, Thermo Fisher). Cell concentration was adjusted to 1,000 cells/μL for 10X Genomics loading.

#### Single-cell library preparation and sequencing

Single-cell suspensions were combined with gel bead-in-emulsion (GEM) reagents using the 10X Genomics Chromium Controller, achieving a target cell-bead co-encapsulation occupancy rate of 0.05 (5%) to optimize droplet formation. Following GEM generation, droplets were reverse-transcribed to synthesize cDNA, and emulsions were subsequently broken to recover individual barcoded transcripts. A 3ʹ gene expression library was constructed through enzymatic fragmentation, end-repair, adapter ligation, and PCR amplification in accordance with the 10X Genomics Chromium Single Cell 3ʹ Reagent Kit protocol. Final libraries were quantified via Qubit fluorometry and Agilent Bioanalyzer quality control, then sequenced on an Illumina NovaSeq 6000 platform (Novogene Co., Ltd., China).

#### Single-cell RNA sequencing data processing and quality control

Raw sequencing data (FASTQ format) from multiple experimental batches were processed through the 10X Genomics Cell Ranger pipeline (v7.1) for alignment against the mouse pre-mRNA reference genome (GRCm38, Ensembl release 102). This workflow generated a UMI-count matrix through default mapping parameters, incorporating cellular barcode and unique molecular identifier annotations. The count matrix was subsequently analyzed using the Seurat^62^ toolkit (v4.1.2) in R, initialized via the Read10X function for data importation. Stringent quality thresholds were applied to filter low-confidence cells: 1. Cells containing fewer than 500 detectable genes (nFeature_RNA <500); 2. Cells exhibiting mitochondrial gene content >20% (percent.mt > 20); 3. Genes detected in fewer than 10 cells (nCount_RNA <10).

#### Data normalization and feature selection

Following quality control, single-cell RNA sequencing datasets were normalized using Seurat’s SCTransform workflow (v5) to mitigate technical variability. Highly variable genes (HVGs) were identified through variance-stabilizing transformation implemented in the FindVariableFeatures algorithm, which prioritizes genes exhibiting dispersion above expected mean-variance relationships. A consensus set of 2,000 HVGs was retained for downstream analysis.

#### Cluster detection

Principal component analysis (PCA) was performed on the normalized data using 2,000 selected variable genes to reduce dimensionality. We identify 40 significant components who have a strong enrichment of low *p* value features to compute the representations using the ‘JackStrawPlot’ function in Seurat. The top 40 principal components were selected for downstream analyses based on the standard deviation explained. Cells were clustered using the Louvain algorithm implemented in the ‘FindClusters’ function, with a resolution of 0.8 set to ensure that distinct cell populations were identified.

#### Dimensional reduction and cellular clustering

The normalized expression matrix underwent dimensionality reduction via principal component analysis (PCA), leveraging the pre-defined set of 2,000 HVGs. Statistically significant principal components (PCs) were determined through permutation testing (JackStraw resampling method, *p* < 0.01), with the top 40 PCs retained for downstream processing based on both significance thresholds and cumulative variance contribution (>90% total variance). Cellular clustering was executed using a graph-based modularity optimization approach (Louvain algorithm) within a shared nearest neighbor (SNN) network derived from PCA embeddings. The clustering resolution parameter was empirically optimized to 0.8 through iterative testing, yielding transcriptionally distinct cell subpopulations while avoiding overpartitioning artifacts.

#### Doublet removal

We used DoubletFinder^63^ (https://github.com/chris-mcginnis-ucsf/DoubletFinder) to identify doublets within each batch, independent of all other datasets. We used 40 principal components (PCs) and a fixed value for the variable numbers of artificial doublets (pN) of 0.25. For the neighborhood size (pK), we iterated over multiple values to find an optimal one for each batch but limited it to a maximum of 0.1.

#### Batch identification

To remove batch effects and integrate the datasets, the following steps were performed: the filtered and normalized Seurat objects from each batch were combined into a list. Canonical Correlation Analysis (CCA) was employed to identify anchors between datasets using the FindIntegrationAnchors function, which was run with dimensionality settings where the first 40 dimensions were used for integration. Datasets were then integrated using the identified anchors through the IntegrateData function. This step produces a new Seurat object containing the integrated dataset with minimized batch effects. The combined datasets were normalized and scaled using the ScaleData function. Principal Component Analysis (PCA) was performed on the scaled data to reduce dimensionality. Subsequently, Uniform Manifold Approximation and Projection (UMAP) was used for further dimensionality reduction and visualization, utilizing the first 40 principal components. Finally, we visualized the integrated data using DimPlot to assess the effectiveness of batch effect removal according to cell-type purity, and cluster separation.

#### Marker gene detection

We used the van Elteren test implementation for Seurat objects (https://github.com/KChen-lab/stratified-tests-for-seurat) to detect marker genes for each node of the cluster tree, comparing the current cluster either against all other nodes on the same level or against the sibling nodes to obtain a set of markers per cluster.

#### Cell type annotation

For cell type annotation, several approaches were employed: known marker genes for each cell type were identified from the literature^28^ or publicly available databases (e.g., CellMarker^68^). Each cluster was manually annotated based on the expression profiles of the marker genes. Cells within a cluster exhibiting high expression of specific marker genes were assigned a corresponding cell type label. After initial annotations, the consistency of cell type assignments was validated through differential expression analysis among clusters, verifying that cluster-specific marker genes reflected expected biological functions. Clustering stability was further confirmed through re-clustering analyses to examine the expression patterns of known cell type-specific genes. The annotations were cross-validated by comparing cluster identities with relevant literature and existing databases. The expression levels of these marker genes were visualized using the FeaturePlot function. The final cell type annotations were confirmed by examining cluster compositions and visualizing UMAP plots colored by cell type assignments.

#### Differentially expressed genes (DEGs) analysis

DEGs between groups were performed using the FindMarkers function of Seurat using the Wilcoxon signed-rank test. Cells with a minimum 90% percentage in either of the two groups of cells were used differential gene expression analysis. To consider a gene as DEGs, it had to meet the following criteria^69^: adjusted *P* adjust values <0.05 and |Log_2_ fold change| > 0.25.

#### Trajectory analysis of scRNA-seq datasets

To reconstruct developmental or differentiation trajectories, we performed pseudotemporal ordering of cells using Slingshot.^64^ The algorithm first constructs a cluster-based minimum spanning tree (MST) using cluster centroids as nodes, establishing an initial topology of cellular transitions. Simultaneous principal curves were then fitted across all branching lineages to model nonlinear pseudotime trajectories while preserving lineage-specific dynamics. Cells were ordered along trajectories based on their transcriptional progression, and trajectory robustness was assessed through bootstrapping (*n* = 100 iterations) to quantify confidence in branchpoint assignments. Results were visualized via UMAP embeddings overlaid with trajectory paths and pseudotime gradients to highlight key transitional states and bifurcation events.

#### Transcriptional regulatory network analysis

We constructed transcriptional regulatory networks using the SCENIC^65^ pipeline (v1.1.2). A reference set of transcription factors (TFs) and their binding motifs was curated from RcisTarget based on the GRCm38 (mm10) genome assembly. Gene regulatory networks (GRNs) were inferred using two complementary algorithms: GENIE3 for context-specific TF-target predictions and GRNBoost for ensemble-based network optimization. Subtype-specific regulatory networks were derived by integrating differentially expressed genes (*p* adjust value <0.05, Log_2_ fold change >0.25) as input constraints to prioritize lineage-defining TFs. To resolve dynamic regulatory changes during differentiation, pseudotime-ordered gene expression trajectories were analyzed through RcisTarget to identify temporally enriched TF binding motifs (normalized enrichment score [NES] > 3.0, FDR <0.05). Final networks were visualized in cytoscape^70^ (v3.8.1) using edge-weighted force-directed layouts and modularity clustering to highlight hub regulators and core subnetworks.

#### Functional annotation analysis

Gene Ontology (GO) enrichment analysis was performed using clusterProfiler^66^ (v4.1.0) to functionally characterize differentially expressed genes (DEGs). Biological processes, molecular functions, and cellular compartments were systematically interrogated to identify pathways enriched in DEGs (|Log_2_ fold change| > 1, *P* adjust value <0.05). Significant GO terms were selected based on a false discovery rate (FDR)-adjusted *p* value threshold of <0.05, with the Benjamini-Hochberg method applied for multiple testing corrections. Results were visualized using R package ggplot2 (v3.0.1), displaying enriched terms as dot plots or bar charts sorted by fold enrichment, with color gradients reflecting statistical significance and node size proportional to gene counts.

#### Gene set enrichment scoring

Gene sets representing biological processes, molecular functions, and cellular components were curated from AmiGO,^71^ the official Gene Ontology knowledgebase. Cellular enrichment scores for these gene sets were calculated using the AddModuleScore function in Seurat, which aggregates normalized expression values of set-specific genes into per-cell scores while controlling for confounding factors. Differential activity of gene sets across experimental groups was statistically evaluated using a two-sided Wilcoxon rank-sum test (implemented in R), with significance thresholds set at FDR-adjusted *p* < 0.05 and a minimum Log_2_ fold change >0.25. Results were visualized as violin plots or ridge plots using ggplot2 to highlight condition-specific pathway activation.

#### Cell-cell communication analysis

To systematically infer cell-cell communication networks, we performed ligand-receptor interaction analysis using CellChat^67^ (v1.1.3). This method integrates a curated database of ligand-receptor pairs to probabilistically model communication pathways between cell types. Signaling interactions were quantified by calculating communication probabilities, and statistically significant interactions (*p* < 0.01, determined through permutation testing) were prioritized for downstream analysis. Key findings were visualized using heatmaps and network diagrams to highlight dominant signaling axes and directional communication patterns.

### Quantification and statistical analysis

Statistical analyses were performed using R software (version 4.1.2). Statistical methods were selected according to the data structure and distributional characteristics of each analysis. For single-cell RNA-seq–related analyses, statistical testing was conducted using the methods implemented in the corresponding analysis packages. These included Wilcoxon-based tests implemented in the Seurat package for differential analyses and gene set enrichment scoring, Benjamini–Hochberg correction for multiple testing in functional annotation analyses, and permutation-based statistical testing implemented in the *CellChat* package for cell–cell communication analyses. For conventional experimental phenotype data, group comparisons were performed using Student’s t-tests when data were approximately normally distributed; otherwise, the Wilcoxon Rank-Sum test was applied. *p*-values are reported in the corresponding figures to indicate statistical significance. The reproducibility of results was verified through independent experimental replicates. Details of the specific statistical tests applied in each analysis are provided in the relevant figure legends. Unless otherwise stated, grouped quantitative data are presented as mean ± SEM. Statistical significance is indicated as ∗*p* < 0.05, ∗∗*p* < 0.01, ∗∗∗*p* < 0.001, and ∗∗∗∗*p* < 0.0001; the statistical test used for each analysis is specified in the corresponding figure legend or Methods subsection.

### Additional resources

This study did not generate additional resources.

## Supplemental information

Document S1. Figures S1–S6 and Data S1

Table S1. Top 100 differentially expressed genes for each cell type after Ara-C treatment

Table S2. Cell numbers for each cell type in each single-cell RNA-seq sample

Table S3. Numbers of differentially expressed genes for each cell type after Ara-C treatment

Table S4. Cell proportions of spermatogonial subtypes by group

Table S5. GO and KEGG enrichment analysis of up-regulated DEGs in spermatogonia

Table S6. GO enrichment analysis of up- and down-regulated DEGs in undifferentiated spermatogonia

Table S7. KEGG pathway enrichment of unique DEGs in elongating spermatids

Table S8. KEGG pathway enrichment across sperm cell types

Table S9. GO enrichment analysis of KLF9-regulated genes

Table S10. KEGG pathway enrichment for Leydig cells

Table S11. qPCR primer sequences used in this study
