# Supplementary material for: Decoding cytarabine-induced damage in the prepubertal testis: A multi-omics atlas of ecosystem and inheritance
Source: iScience. 2026 Aug 5;29(8):117094. doi: 10.1016/j.isci.2026.117094 (PMC13474082; doi:10.1016/j.isci.2026.117094)
Supplement: Supplementary file 1 [file mmc1.pdf]

iScience, Volume *29*

Supplemental information

Decoding cytarabine-induced damage in the prepubertal testis: A multi-omics atlas of ecosystem and inheritance

Siyu Xia, Jiangpeng Wu, Jiawei Duan, Wenting Ye, Yan Sun, Xiaojian Yang, Xiao Chen, Hongru Lin, Songyu Huang, Jing Cai, Haiping Wen, and Yali Song

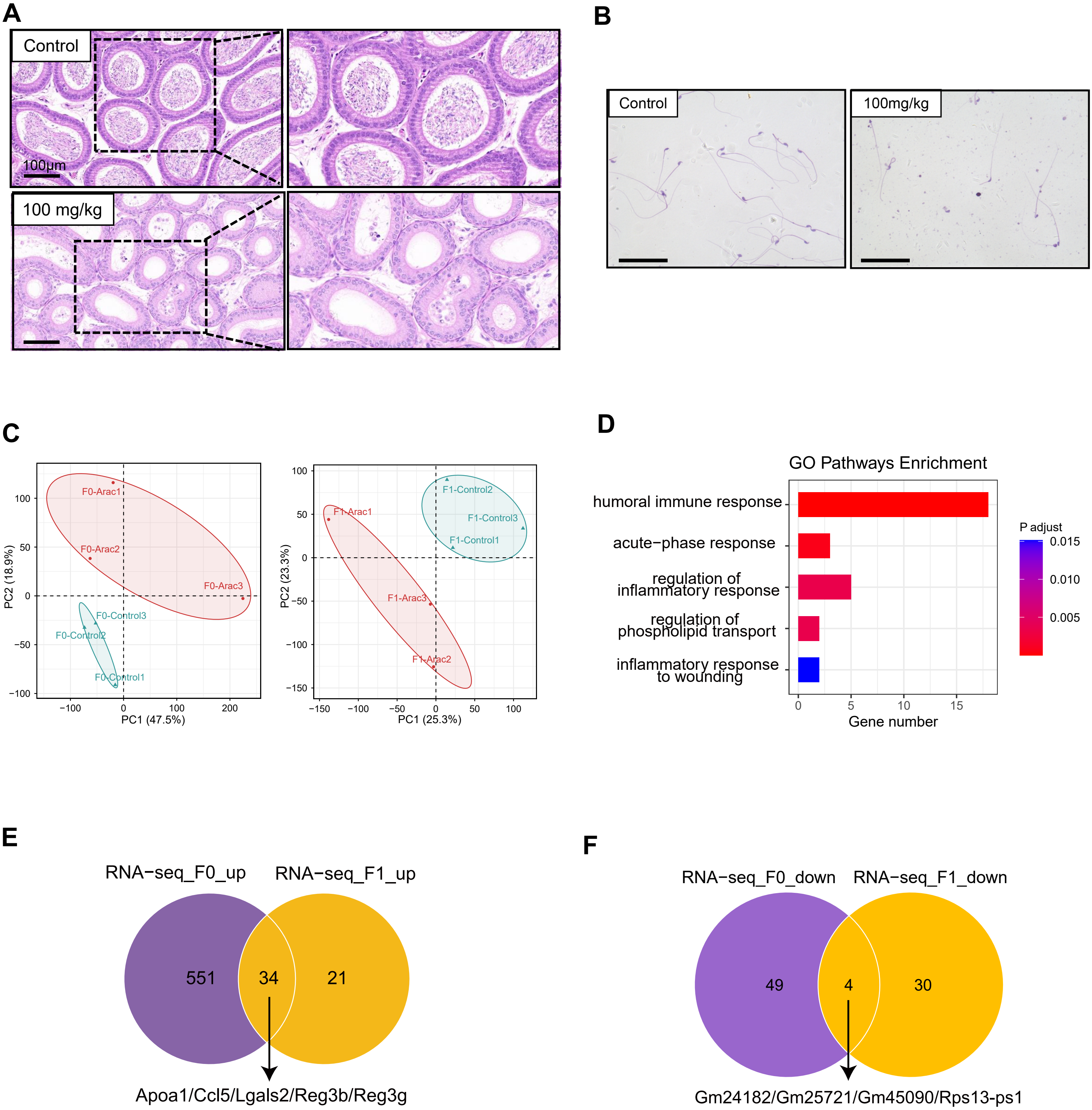

A
Control
100μm
100 mg/kg
B
Control
100mg/kg
C
F0-Arac1
F0-Arac2
F0-Arac3
F0-Control3
F0-Control2
F0-Control1
PC2 (18.9%)
PC1 (47.5%)
F1-Control2
F1-Control3
F1-Control1
F1-Arac1
F1-Arac3
F1-Arac2
PC2 (23.3%)
PC1 (25.3%)
D
GO Pathways Enrichment
humoral immune response
acute−phase response
regulation of inflammatory response
regulation of phospholipid transport
inflammatory response to wounding
Gene number
P adjust
0.015
0.010
0.005
E
RNA−seq_F0_up
RNA−seq_F1_up
551
34
21
Apoa1/Ccl5/Lgals2/Reg3b/Reg3g
F
RNA−seq_F0_down
RNA−seq_F1_down
49
4
30
Gm24182/Gm25721/Gm45090/Rps13-ps1

Figure S1.

Morphological and intergenerational transcriptional effects of prepubertal Ara-C exposure. (A–B) Representative H&E images of cauda epididymis from control and 100 mg/kg Ara-C–treated mice showing epithelial exfoliation, degeneration, and decreased sperm density; quantification confirms significant sperm reduction. Shown are representative images from each group (n = 3 biologically independent animals). Scale bars: 100 um. (C) PCA plot showing distinct transcriptomic clustering between control and Ara-C–treated F0 testes, and between their respective F1 offspring. (D) Bar plot showing GO pathway enrichment of F1-specific DEGs; bar length represents gene number, and color intensity (blue to red) reflects adjusted P values. (E-F) Veen plot displaying persistently dysregulated immune and inflammatory genes (e.g., Apoa1, Ccl5) across generations.

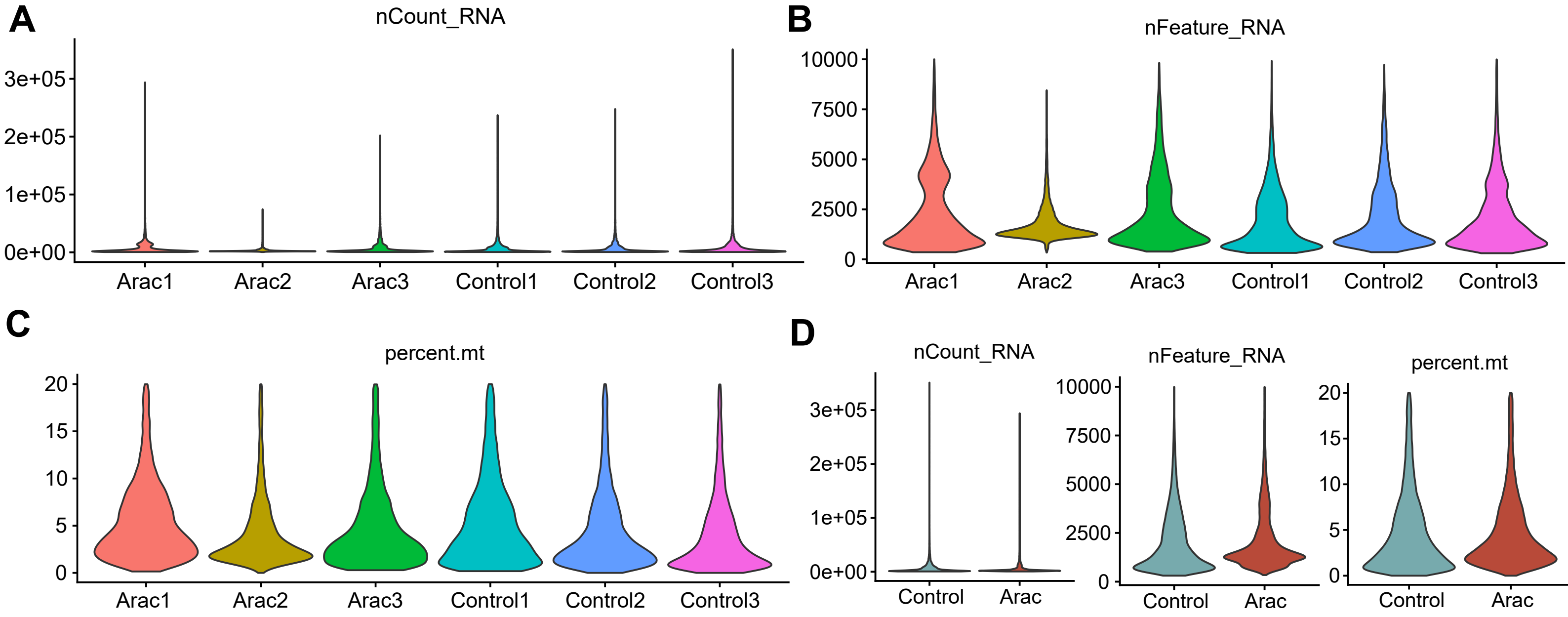

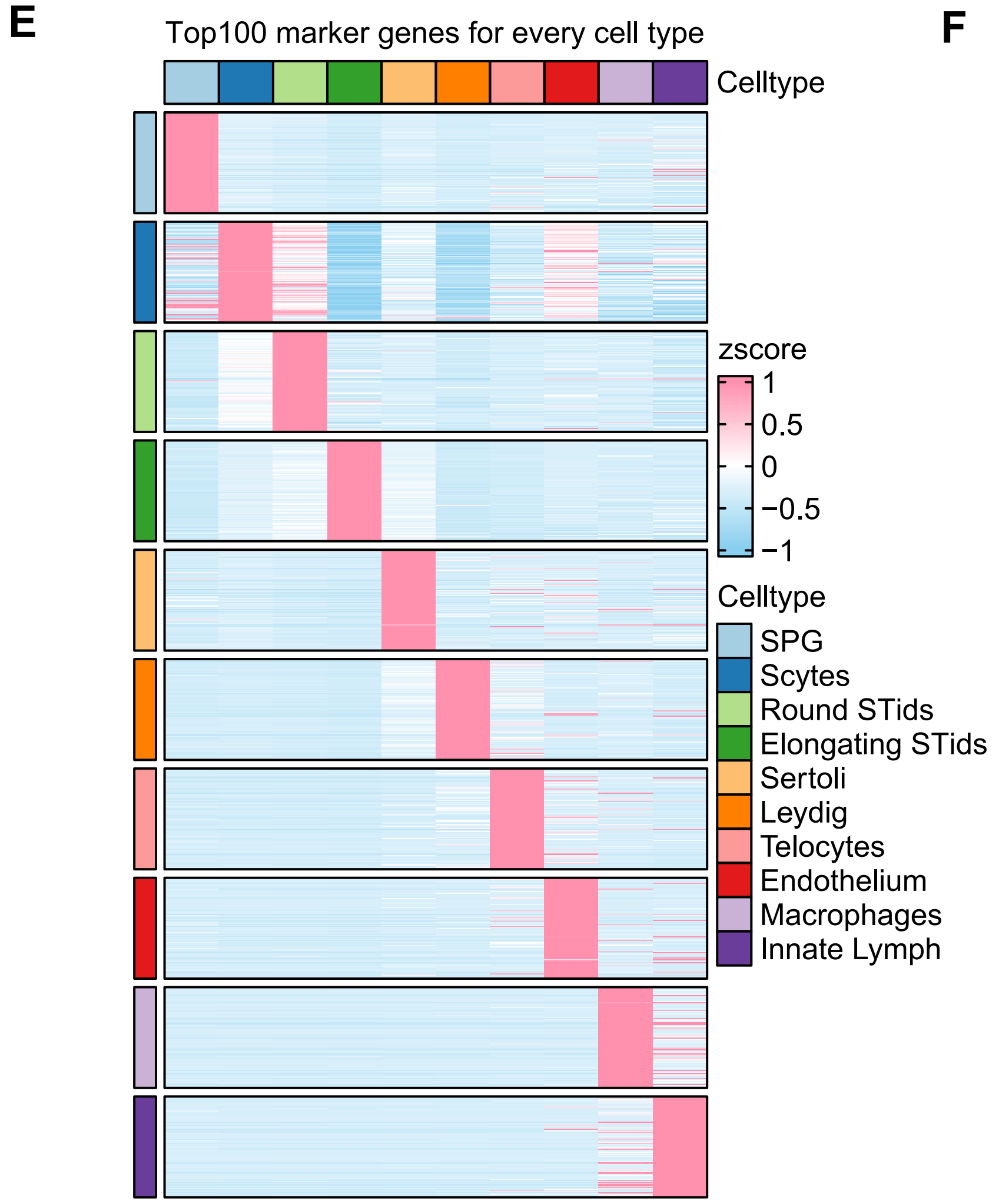

F

The number of DEGs for each cell type after Arac treatment

Celltype	DEGs	Change
SPG	807	Upregulated
	2056	Downregulated
Scytes	548	Upregulated
	461	Downregulated
Round STids	1231	Upregulated
	704	Downregulated
Elongating STids	3363	Upregulated
	340	Downregulated
Sertoli	876	Upregulated
	390	Downregulated
Leydig	2871	Upregulated
	395	Downregulated
Telocytes	291	Upregulated
	82	Downregulated
Endothelium	729	Upregulated
	79	Downregulated
Macrophages	149	Upregulated
	63	Downregulated
Innate Lymph	6	Upregulated
	9	Downregulated

G

The cell number of each cell type for every sample

Cell Type / Sample	Control1	Control2	Control3	Arac1	Arac2	Arac3
SPG	926	489	1418	490	654	168
Scytes	3869	3500	2240	2414	4766	2896
Round STids	2158	1928	2027	2233	4353	2998
Elongating STids	2738	1734	2878	564	271	757
Sertoli	2799	613	373	534	385	500
Leydig	1483	738	596	1544	1230	919
Telocytes	220	245	387	765	444	111
Endothelium	370	257	47	97	374	110
Macrophages	129	75	269	407	107	29
Innate Lymph	83	25	26	33	17	3

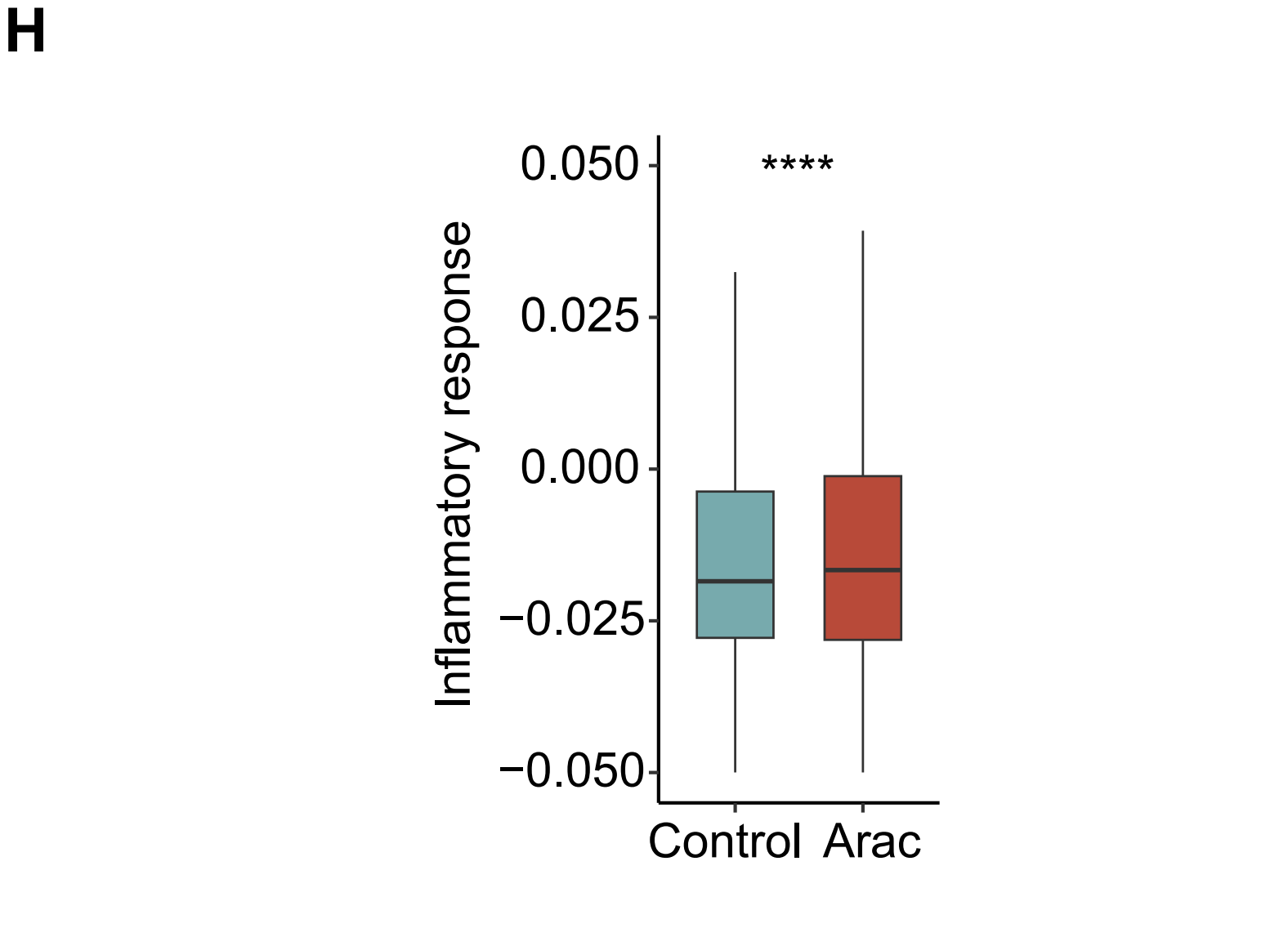

Figure S2.

Quality control and validation of single-cell RNA-seq data from Ara-C–treated testes. (A–D) QC plots showing cell-level filtering metrics, including gene counts, UMI counts, and mitochondrial gene percentages, after quality control. (E) Heatmap displaying expression of canonical marker genes across clusters, confirming accurate annotation of nine distinct testicular cell populations. (F) Table graph comparing DEG counts (up- and down-regulated) across cell types under Ara-C exposure, showing the largest upregulation in Elongating STids and predominant downregulation in SPG. (G) Table graph showing absolute cell type composition in control and Ara-C–treated testes. (H) Gene set score analysis of pathways related to inflammatory response, demonstrating activation across all cell types following Ara-C exposure. Statistical significance determined by two-sided Wilcoxon rank-sum tests: *P < 0.05; **P < 0.01; ***P < 0.001; ***P < 0.0001.

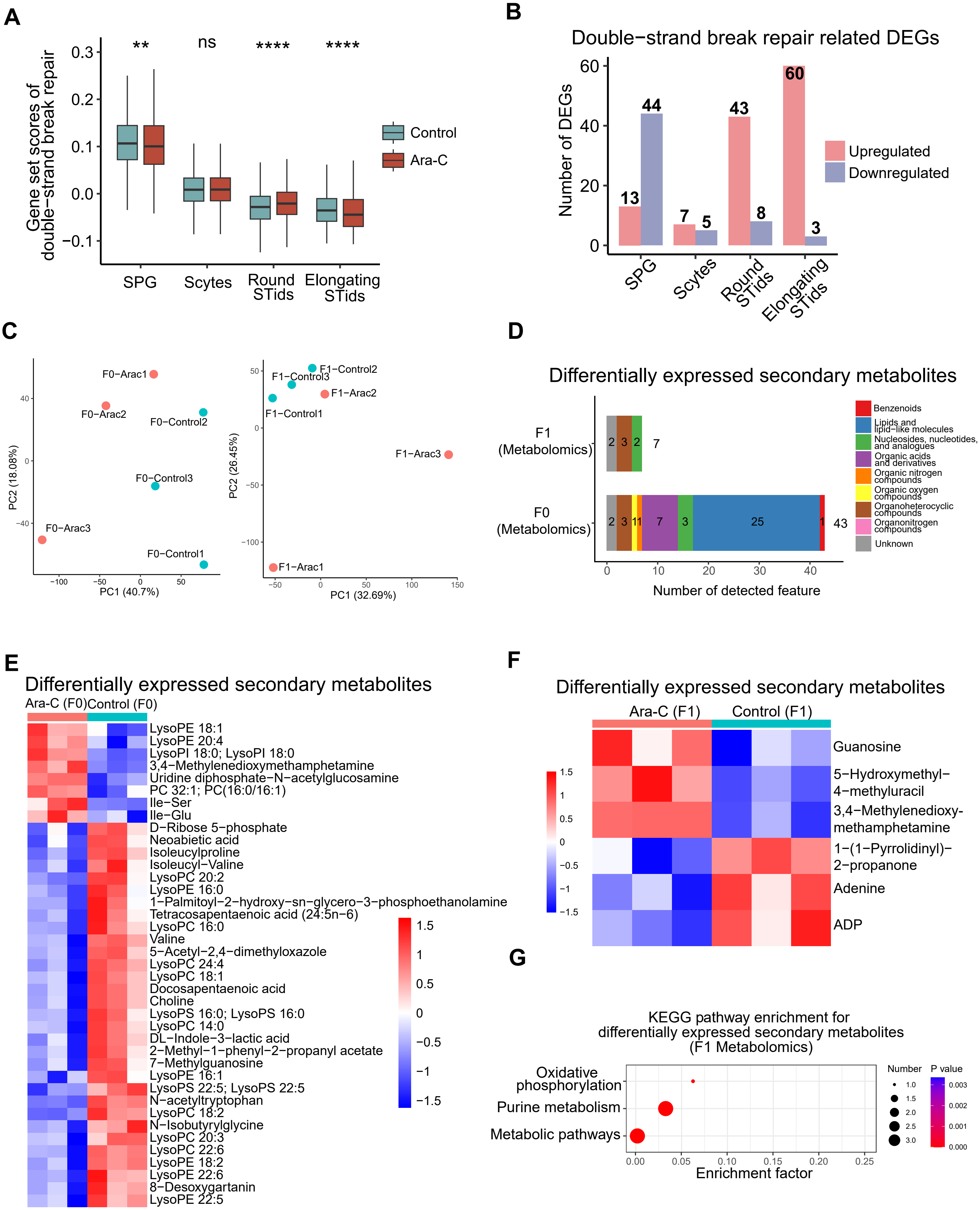
A
Gene set scores of double−strand break repair
0.3
0.2
0.1
0.0
−0.1
**
ns
****
****
Control
Ara-C
SPG
Scytes
Round STids
Elongating STids
B
Double−strand break repair related DEGs
Number of DEGs
60
40
20
0
13
44
7
5
43
8
60
3
Upregulated
Downregulated
SPG
Scytes
Round STids
Elongating STids
C
F0−Arac1
F0−Arac2
F0−Control2
F0−Control3
F0−Arac3
F0−Control1
PC2 (18.08%)
PC1 (40.7%)
F1−Control2
F1−Control3
F1−Arac2
F1−Control1
F1−Arac3
F1−Arac1
PC2 (26.45%)
PC1 (32.69%)
D
Differentially expressed secondary metabolites
F1 (Metabolomics)
F0 (Metabolomics)
Number of detected feature
Benzenoids
Lipids and lipid−like molecules
Nucleosides, nucleotides, and analogues
Organic acids and derivatives
Organic nitrogen compounds
Organic oxygen compounds
Organoheterocyclic compounds
Organonitrogen compounds
Unknown
E
Differentially expressed secondary metabolites
Ara-C (F0)
Control (F0)
LysoPE 18:1
LysoPE 20:4
LysoPI 18:0; LysoPI 18:0
3,4−Methylenedioxymethamphetamine
Uridine diphosphate−N−acetylglucosamine
PC 32:1; PC(16:0/16:1)
Ile−Ser
Ile−Glu
D−Ribose 5−phosphate
Neoabietic acid
Isoleucylproline
Isoleucyl−Valine
LysoPC 20:2
LysoPE 16:0
1−Palmitoyl−2−hydroxy−sn−glycero−3−phosphoethanolamine
Tetracosapentaenoic acid (24:5n−6)
LysoPC 16:0
Valine
5−Acetyl−2,4−dimethyloxazole
LysoPC 24:4
LysoPC 18:1
Docosapentaenoic acid
Choline
LysoPS 16:0; LysoPS 16:0
LysoPC 14:0
DL−Indole−3−lactic acid
2−Methyl−1−phenyl−2−propanyl acetate
7−Methylguanosine
LysoPE 16:1
LysoPS 22:5; LysoPS 22:5
N−acetyltryptophan
LysoPC 18:2
N−Isobutyrylglycine
LysoPC 20:3
LysoPC 22:6
LysoPE 18:2
LysoPE 22:6
8−Desoxygartanin
LysoPE 22:5
F
Differentially expressed secondary metabolites
Ara-C (F1)
Control (F1)
Guanosine
5−Hydroxymethyl−4−methyluracil
3,4−Methylenedioxy-methamphetamine
1−(1−Pyrrolidinyl)−2−propanone
Adenine
ADP
G
KEGG pathway enrichment for differentially expressed secondary metabolites (F1 Metabolomics)
Oxidative phosphorylation
Purine metabolism
Metabolic pathways
Enrichment factor
Number
P value

Figure S3.

Transcriptomic and metabolic evidence for Ara-C–induced stress response and metabolic remodeling. (A–B) Gene set score analysis of DNA double-strand break repair pathways showing marked inhibition in both SPG and Elongating STids, with polarized distribution of repair-related gene expression. Statistical analysis by two-sided Wilcoxon rank-sum test: *P < 0.05; **P < 0.01; ***P < 0.001; ***P < 0.0001 vs. control. (C) PCA plots showing clear separation between control and Ara-C groups in both F0 and F1 testes metabolomes, indicating robust Ara-C–induced metabolic remodeling. (D) Bar plot showing metabolic pathway enrichment of altered metabolites in F0 and F1 generations. The F0 generation exhibited broad enrichment across lipid, amino acid, and nucleotide metabolism, while F1 enrichment was mainly restricted to purine metabolism and oxidative phosphorylation. (E–G) Heatmaps showing differential metabolite abundance in testes of F0 and F1 generations. F0 testes displayed widespread alterations in membrane lipids (LysoPC, LysoPE, LysoPS), amino acids, dipeptides, and nucleotides, whereas F1 changes were largely confined to nucleotide derivatives (guanosine, adenine, ADP).

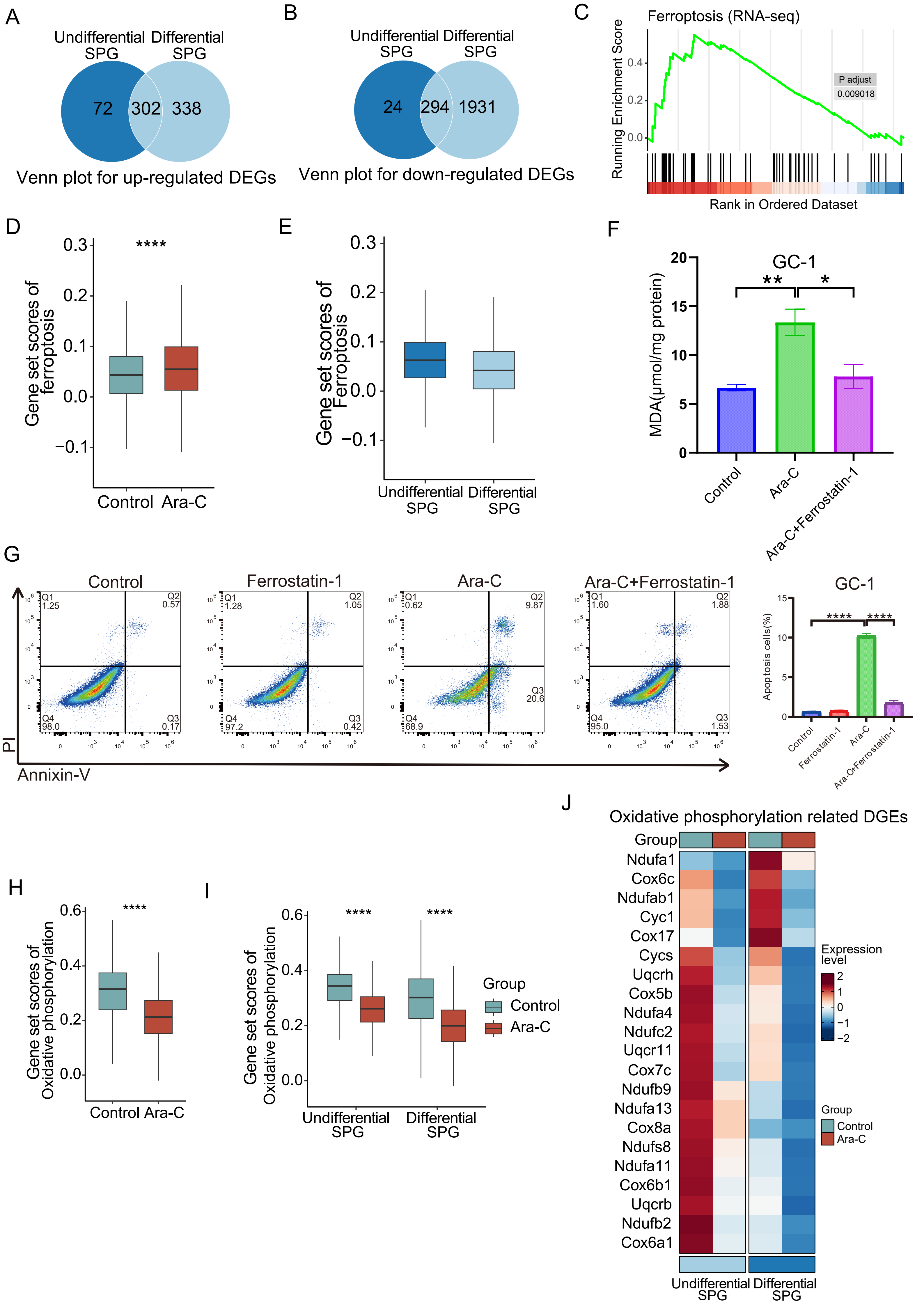

A
Undifferential SPG
Differential SPG
72
302
338
Venn plot for up-regulated DEGs
B
Undifferential SPG
Differential SPG
24
294
1931
Venn plot for down-regulated DEGs
C
Ferroptosis (RNA-seq)
Running Enrichment Score
P adjust
0.009018
Rank in Ordered Dataset
D
Gene set scores of ferroptosis
****
Control
Ara-C
E
Gene set scores of Ferroptosis
Undifferential SPG
Differential SPG
F
GC-1
MDA(μmol/mg protein)
**
*
Control
Ara-C
Ara-C+Ferrostatin-1
G
Control
Ferrostatin-1
Ara-C
Ara-C+Ferrostatin-1
PI
Annixin-V
Q1 1.25
Q2 0.57
Q4 98.0
Q3 0.17
Q1 1.28
Q2 1.05
Q4 97.2
Q3 0.42
Q1 0.62
Q2 9.87
Q4 68.9
Q3 20.6
Q1 1.60
Q2 1.88
Q4 95.0
Q3 1.53
GC-1
Apoptosis cells(%)
****
****
Control
Ferrostatin-1
Ara-C
Ara-C+Ferrostatin-1
H
Gene set scores of Oxidative phosphorylation
****
Control
Ara-C
I
Gene set scores of Oxidative phosphorylation
****
****
Undifferential SPG
Differential SPG
Group
Control
Ara-C
J
Oxidative phosphorylation related DGEs
Group
Ndufa1
Cox6c
Ndufab1
Cyc1
Cox17
Cycs
Uqcrh
Cox5b
Ndufa4
Ndufc2
Uqcr11
Cox7c
Ndufb9
Ndufa13
Cox8a
Ndufs8
Ndufa11
Cox6b1
Uqcrb
Ndufb2
Cox6a1
Expression level
Group
Control
Ara-C
Undifferential SPG
Differential SPG

Figure S4.

Transcriptional heterogeneity and ferroptotic and mitochondrial dysregulation in Ara-C–treated spermatogonia. (A–B) Venn diagrams showing overlap and distribution of DEGs between undifferentiated and differentiated SPG, with most DEGs being cluster-specific. (C) Gene set enrichment analysis (GSEA) from bulk RNA-seq data showing significant activation of ferroptosis pathways in Ara-C–treated testes. (D–E) Gene set scores analysis demonstrating ferroptosis activation in both undifferentiated and differentiated SPG clusters. Statistical analysis by two-sided Wilcoxon rank-sum test: *P < 0.05; **P < 0.01; ***P < 0.001; ***P < 0.0001 vs. control. (F) Bar plot showing that malondialdehyde (MDA) was significantly elevated upon treatment with Ara-C, validating ferroptosis activation in SPG, whereas Ferrostatin-1 (Fer-1), a potent inhibitor of ferroptosis, partially reversed these changes. Data are presented as mean +/- SEM (n = 6 independent replicates). Statistical significance determined by Student's t-test: *P < 0.05; **P < 0.01; ***P < 0.001; ****P < 0.0001 vs. control. (G) Representative flow cytometry plots of Annexin V/PI staining in GC-1 SPG cells treated with vehicle, Ara-C, or Ara-C combined with the ferroptosis inhibitor Ferrostatin-1 (Fer-1, 0.5 uM). Bar plot quantifying the apoptosis rate, showing that Fer-1 significantly ameliorates Ara-C–induced cell death. Data are presented as mean +/- SEM of n = 3 independent biological replicates. Statistical significance determined by Student's t-test: *P < 0.05; **P < 0.01; ***P < 0.001; ****P < 0.0001 vs. control. (H-I) Bar plots showing consistent repression of oxidative phosphorylation. Statistical analysis by two-sided Wilcoxon rank-sum test: *P < 0.05; **P < 0.01; ***P < 0.001; ****P < 0.0001 vs. control. (J) Heatmap showing differentially expressed genes (DEGs) associated with oxidative phosphorylation.

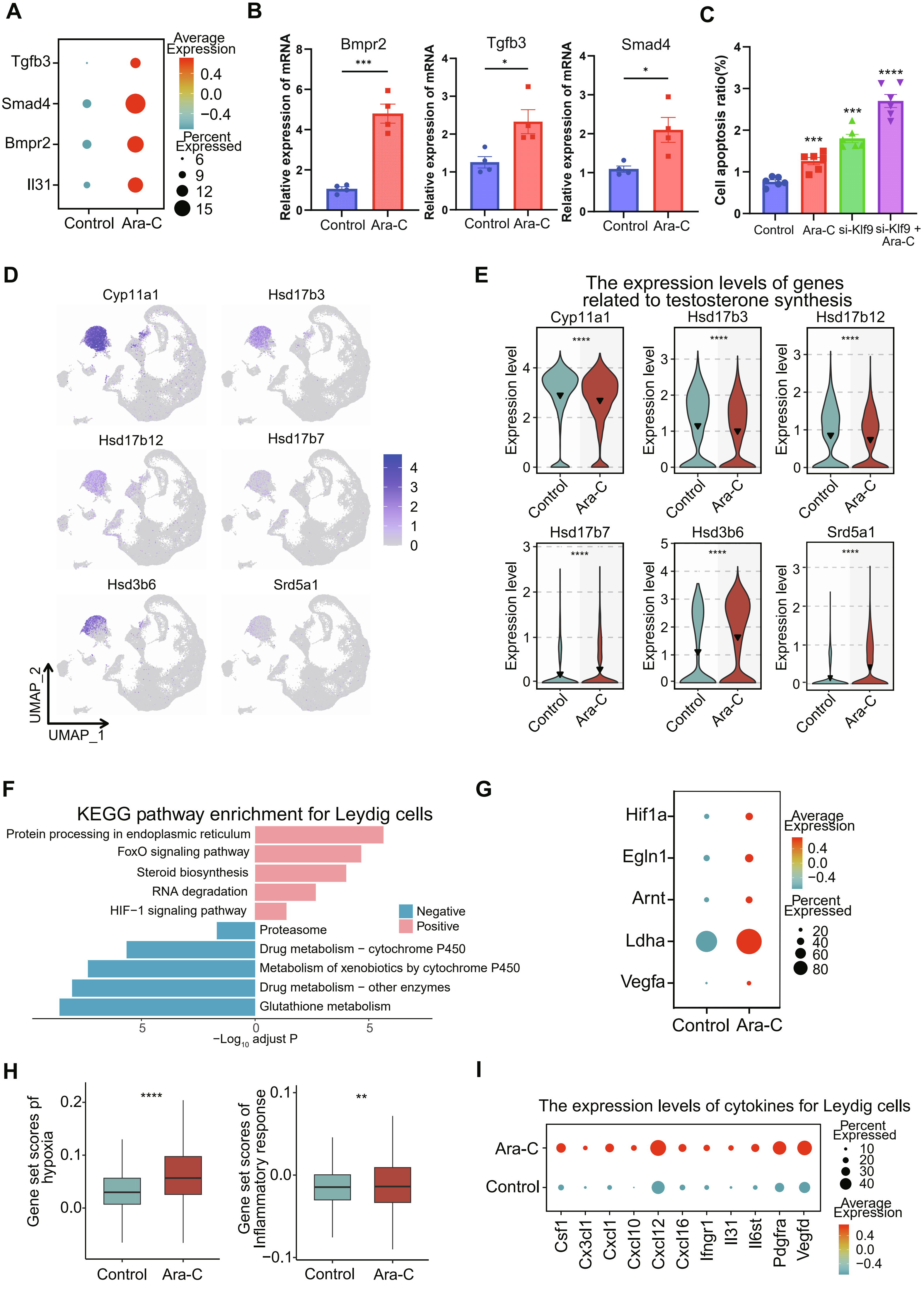

A
Average Expression
0.4
0.0
−0.4
Percent Expressed
6
9
12
15
Tgfb3
Smad4
Bmpr2
Il31
Control
Ara-C
B
Bmpr2
Tgfb3
Smad4
Relative expression of mRNA
***
*
*
Control
Ara-C
C
Cell apoptosis ratio(%)
***
***
****
Control
Ara-C
si-Klf9
si-Klf9 + Ara-C
D
Cyp11a1
Hsd17b3
Hsd17b12
Hsd17b7
Hsd3b6
Srd5a1
UMAP_2
UMAP_1
E
The expression levels of genes related to testosterone synthesis
Cyp11a1
Hsd17b3
Hsd17b12
Hsd17b7
Hsd3b6
Srd5a1
Expression level
****
Control
Ara-C
F
KEGG pathway enrichment for Leydig cells
Protein processing in endoplasmic reticulum
FoxO signaling pathway
Steroid biosynthesis
RNA degradation
HIF−1 signaling pathway
Proteasome
Drug metabolism − cytochrome P450
Metabolism of xenobiotics by cytochrome P450
Drug metabolism − other enzymes
Glutathione metabolism
Negative
Positive
−Log10 adjust P
G
Hif1a
Egln1
Arnt
Ldha
Vegfa
Average Expression
Percent Expressed
20
40
60
80
Control
Ara-C
H
Gene set scores pf hypoxia
****
Gene set scores of Inflammatory response
**
Control
Ara-C
I
The expression levels of cytokines for Leydig cells
Ara-C
Control
Csf1
Cx3cl1
Cxcl1
Cxcl10
Cxcl12
Cxcl16
Ifngr1
Il31
Il6st
Pdgfra
Vegfd
Percent Expressed
10
20
30
40
Average Expression

Figure S5.

KLF9-regulated stress adaptation in Sertoli cells and Ara-C–induced testosterone suppression and inflammatory activation in Leydig cells. (A) Heatmap demonstrating activation of the TGFβ/BMP signaling cascade in Ara-C–treated Sertoli cells, with upregulation of Tgfb3, Smad4 and Bmpr2. (B) qPCR validation of TGFβ/BMP and inflammatory mediators, including Tgfb3, Bmpr2 and Il31, confirming single-cell RNA-seq findings. Data are presented as mean +/- SEM of n = 4 independent biological replicates. Statistical analysis by Student's t-test: *P < 0.05; **P < 0.01; ***P < 0.001; ****P < 0.0001 vs. control. (C) Bar plot quantifying enhanced apoptosis in Klf9-knockdown Sertoli cells exposed to Ara-C. Data are presented as mean +/- SEM of n = 6 independent biological replicates. Statistical analysis by Student's t-test: *P < 0.05; **P < 0.01; ***P < 0.001; ****P < 0.0001 vs. control. (D) UMAP plot showing specific expression of steroidogenic enzymes in Leydig cells. (E) Violin plots showing that key enzymes required for testosterone biosynthesis (Cyp11a1, Hsd17b3, Hsd17b12) were markedly downregulated, whereas intermediate cholesterol metabolism enzymes (Hsd17b7, Hsd3b6, Srd5a1) were upregulated following Ara-C exposure. Statistical significance determined by two-sided Wilcoxon rank-sum test: *P < 0.05; **P < 0.01; ***P < 0.001; ****P < 0.0001 vs. control. (F) Bar plot of KEGG pathway enrichment analysis revealing activation of HIF-1 signaling, protein processing in the endoplasmic reticulum, and FoxO signaling, alongside suppression of glutathione and drug metabolism pathways, indicative of oxidative stress and reduced detoxification capacity. Red and blue colors represent positive and negative enrichment scores, respectively. (G) Heatmap of hypoxia-related genes (Hif1a, Egln1, Arnt, Ldha, Vegfa) showing strong induction, consistent with elevated hypoxia, inflammatory response, and cytokine gene set scores in Ara-C–treated Leydig cells. (H) Gene set score analysis showing significantly increased hypoxia and inflammatory response activity, reflecting enhanced immunoregulatory engagement of Leydig cells under stress. Statistical significance determined by two-sided Wilcoxon rank-sum test: *P < 0.05; **P < 0.01; ***P < 0.001; ****P < 0.0001 vs. control. (I) Dot plot of cytokine and growth factor expression profiles revealing robust upregulation of chemokines (Cxcl1, Cxcl10, Cxcl12, Cxcl16), immune receptors (Ifngr1, Il6st), and growth factors (Vegfd, Pdgfra). Dot color represents the average expression level (red: high, blue: low), and dot size represents the percentage of expressing cells.

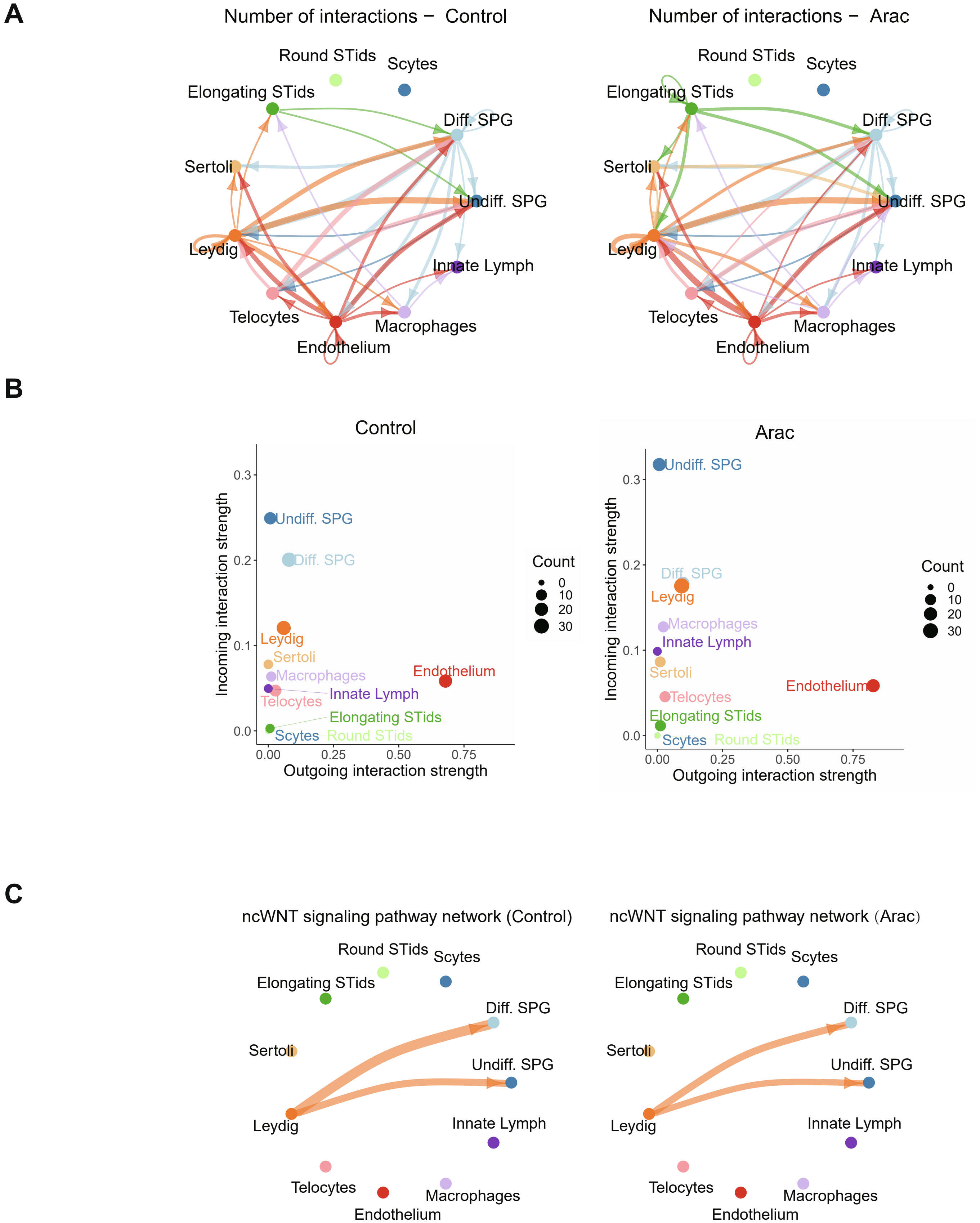
A
Number of interactions − Control
Number of interactions − Arac
Round STids
Scytes
Elongating STids
Diff. SPG
Sertoli
Undiff. SPG
Leydig
Innate Lymph
Telocytes
Macrophages
Endothelium
B
Control
Arac
Incoming interaction strength
Outgoing interaction strength
Count
0
10
20
30
0.0
0.1
0.2
0.3
0.00
0.25
0.50
0.75
C
ncWNT signaling pathway network (Control)
ncWNT signaling pathway network (Arac)

Figure S6.

Cell-type-specific alterations in communication strength and auxiliary pathway remodeling under Ara-C exposure. (A–B) Network plots visualizing global changes in cell-cell communication. (A) The number and (B) the strength of ligand-receptor interactions is shown for all major testicular cell types. Edge thickness corresponds to the magnitude of change (thicker edges indicate a greater increase in interaction number or strength in the Ara-C group compared to control). (C) Network plot of the non-canonical WNT (ncWNT) signaling pathway under Ara-C treatment. The plot highlights a specific suppression of communication signals (thinner or absent edges) from Leydig cells to both undifferentiated and differentiated spermatogonia (SPG), indicating a targeted disruption in this niche signaling axis.

Data S1.

Raw western blot images supporting Figure 5J-K.

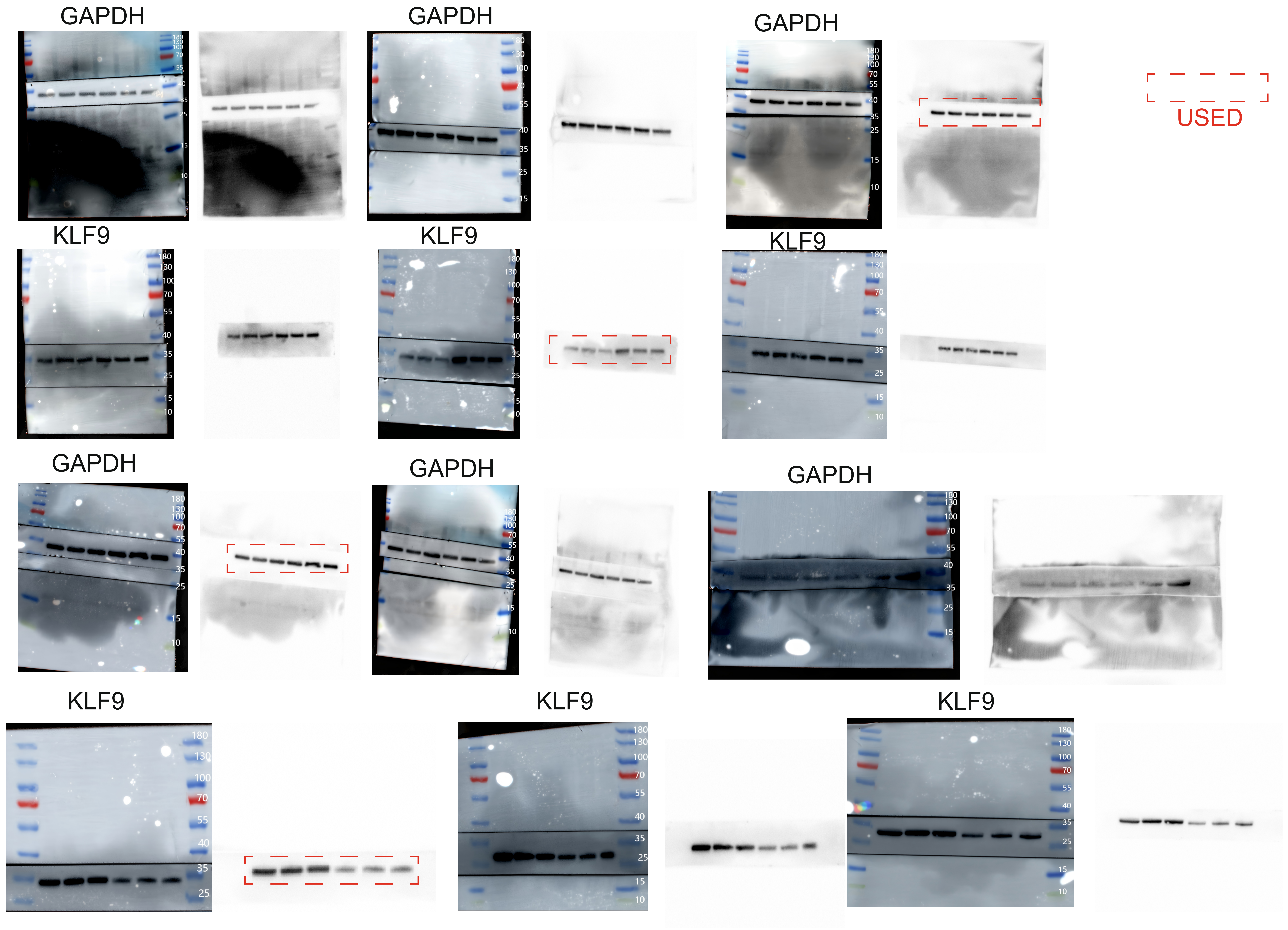
GAPDH
GAPDH
GAPDH
USED
KLF9
KLF9
KLF9
GAPDH
GAPDH
GAPDH
KLF9
KLF9
KLF9

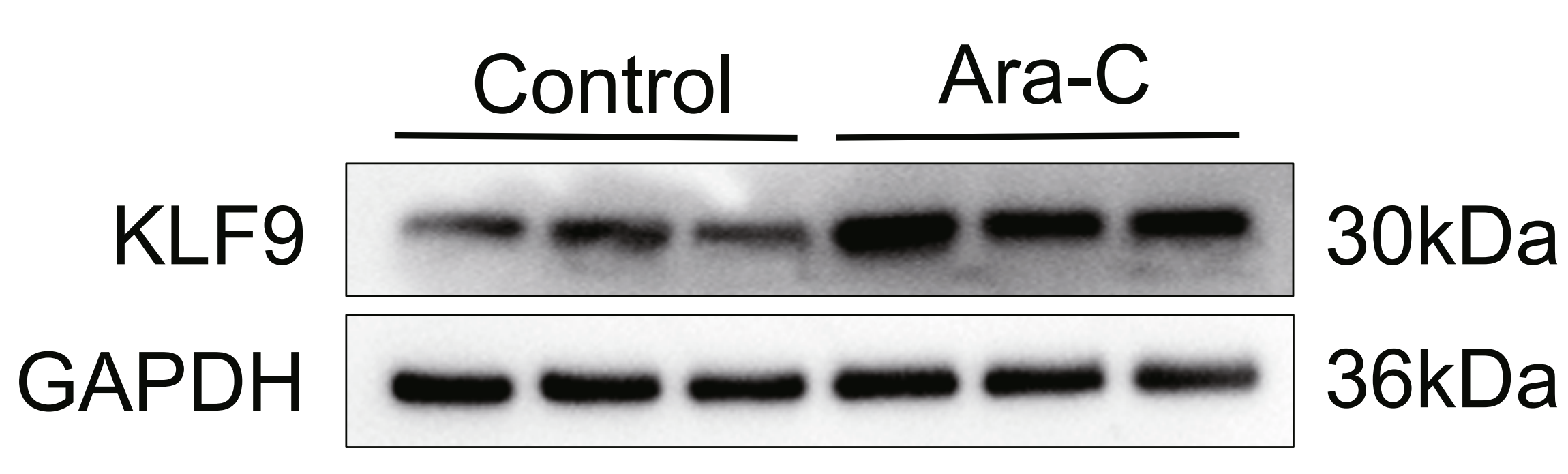
Control
Ara-C
KLF9
30kDa
GAPDH
36kDa

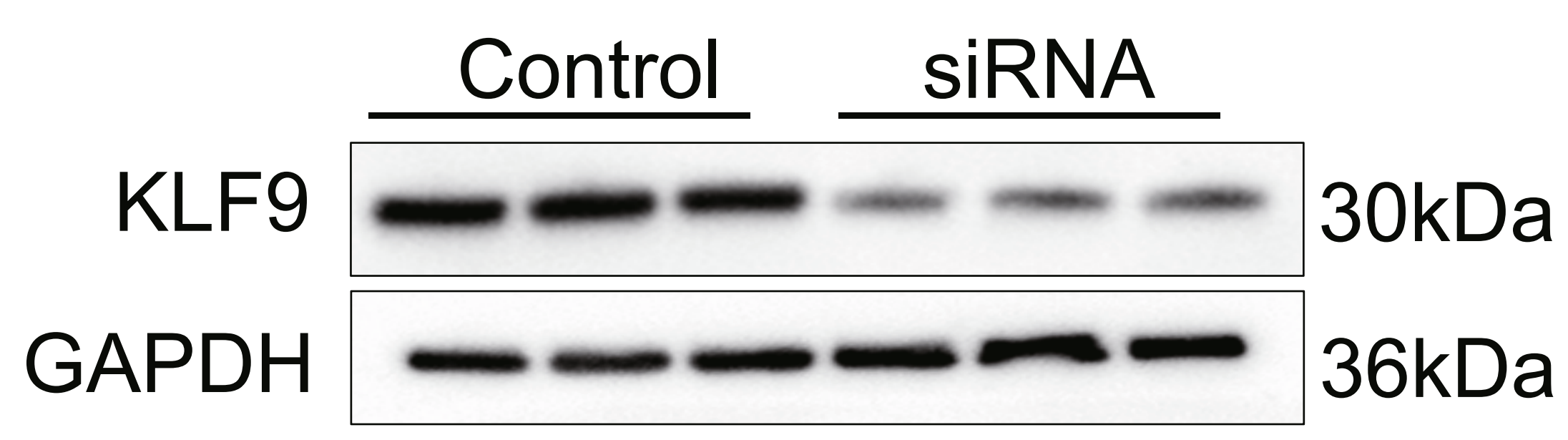
Control
siRNA
KLF9
30kDa
GAPDH
36kDa
